# Silicone cryogel skeletons enhance the survival and mechanical integrity of hydrogel-encapsulated cell therapies

**DOI:** 10.1126/sciadv.adk5949

**Published:** 2024-04-05

**Authors:** William J. Jeang, Matthew A. Bochenek, Suman Bose, Yichao Zhao, Bryan M. Wong, Jiawei Yang, Alexis L. Jiang, Robert Langer, Daniel G. Anderson

**Affiliations:** ^1^ Department of Materials Science and Engineering, Massachusetts Institute of Technology, Cambridge, MA 02139, USA.; ^2^ David H Koch Institute for Integrative Cancer Research, Massachusetts Institute of Technology, Cambridge, MA 02139, USA.; ^3^ Department of Anesthesiology, Critical Care and Pain Medicine, Boston Children’s Hospital, Boston, MA 02115, USA.; ^4^ Department of Chemical Engineering, Massachusetts Institute of Technology, Cambridge, MA 02139, USA.; ^5^ Department of Physiology and Biomedical Engineering, Mayo Clinic, Scottsdale, AZ 85259, USA.; ^6^ Department of Biological Engineering, Massachusetts Institute of Technology, Cambridge, MA 02139, USA.; ^7^ Department of Mechanical and Materials Engineering, Worcester Polytechnic Institute, Worcester, MA 01609, USA.; ^8^ Department of Computer Science, Wellesley College, Wellesley, MA 02481, USA.; ^9^ Institute of Medical Engineering and Science, Massachusetts Institute of Technology, Cambridge, MA 02142, USA.; ^10^ Harvard-MIT Program in Health Sciences and Technology, Massachusetts Institute of Technology, Cambridge, MA 02139, USA.

## Abstract

The transplantation of engineered cells that secrete therapeutic proteins presents a promising method for addressing a range of chronic diseases. However, hydrogels used to encase and protect non-autologous cells from immune rejection often suffer from poor mechanical properties, insufficient oxygenation, and fibrotic encapsulation. Here, we introduce a composite encapsulation system comprising an oxygen-permeable silicone cryogel skeleton, a hydrogel matrix, and a fibrosis-resistant polymer coating. Cryogel skeletons enhance the fracture toughness of conventional alginate hydrogels by 23-fold and oxygen diffusion by 2.8-fold, effectively mitigating both implant fracture and hypoxia of encapsulated cells. Composite implants containing xenogeneic cells engineered to secrete erythropoietin significantly outperform unsupported alginate implants in therapeutic delivery over 8 weeks in immunocompetent mice. By improving mechanical resiliency and sustaining denser cell populations, silicone cryogel skeletons enable more durable and miniaturized therapeutic implants.

## Teaser

Oxygen-permeable skeletons breathe life into implanted cell therapies.

## INTRODUCTION

The transplantation of cells engineered to secrete drugs of interest offers an attractive approach to treating a range of chronic diseases, including autoimmune, liver, neurological, and blood diseases (*1*–*3*). However, direct transplantation of foreign cells can lead to transplant rejection and/or graft-versus-host disease, thus often requiring coadministration of immunosuppressive drugs (*4*, *5*). To circumvent this issue, therapeutic cells may be contained in a device that protects them from the immune system (*6*). A common embodiment of this method involves encapsulating cells in a nondegradable hydrogel (*7*, *8*).

Alginate is one of the most commonly explored hydrogels for encapsulation, due in part to its biocompatibility and ability to cross-link in cell-friendly ionic conditions (*9*–*14*). However, the low toughness of alginate gels and their tendency to swell under physiological conditions make macroscale devices prone to mechanical failure (*15*–*17*). A current strategy to strengthen alginate is to replace calcium with barium as the ionic cross-linker (*18*). Unfortunately, the toxicity of barium ions and their tendency to leach out of gels limit the amount of alginate that can be safely transplanted (*19*). Additional chemical routes such as covalent cross-linking (*20*, *21*), copolymerization (*22*, *23*), and interpenetrating networks (*24*) have been developed to increase hydrogel mechanical properties. However, the concomitant effects of such modifications on biocompatibility, mechanobiology, and mass transport properties can negatively affect both the function of encapsulated cells and the immune response to the implant material (*18*, *25*–*27*). To decouple device mechanics from the hydrogel chemistry, other reports propose physically reinforcing hydrogels by integrating solid plastic constructs such as suture-based fiber cores (*28*) or external housings based on nanoporous membranes and microfabricated housings (*29*–*32*). Nevertheless, fiber-based devices face issues of shape integrity under compression, while external housings create additional diffusion barriers for oxygen delivery to encapsulated cells (*33*–*35*).

A key challenge to the survival of encapsulated cells is the adequate supply of oxygen (*36*). Since encapsulated cells are isolated from the host’s bloodstream, the ambient oxygen level is diminished and requires diffusion for oxygen transport. In addition, the foreign body response (FBR) can lead to the formation of dense fibrous tissue around implants, further hampering transport (*37*–*40*). Strategies to improve the oxygenation of devices include developing antifibrotic materials (*29*, *39*, *41*, *42*), incorporating particles that chemically decompose to release oxygen (*43*–*46*), or supplementing oxygen delivery through transcutaneous gas ports (*47*, *48*). While each of these strategies has shown beneficial impacts on macroencapsulated cell viability, the reliance on exhaustible particles and gas tanks requires routine device replacement or refilling.

Alternative approaches that obviate the use of consumable components exploit materials with enhanced transport properties to efficiently distribute the limited supply of extravascular oxygen. For example, several reports use perfluorocarbon fluids to enhance oxygen diffusion in alginate by incorporating them in emulsions (*49*, *50*). However, such emulsions also can lead to cytotoxicity and a significant decrease in mechanical strength (*50*, *51*). Recently, researchers demonstrated impressive improvements in oxygen transport in hydrogels by using a scaffold formed from plastic containing microscale air pockets (*52*). However, the scaffold contributed more than 70% of the volume of the entire device, requiring a higher density of cells in the hydrogel phase and an overall large device footprint to deliver a therapeutic dosage of cells.

In this study, we leverage the superior gas permeability of silicone elastomers (*53*) and advances in macroporous cryogel synthesis (*54*) to develop multifunctional skeletal structures for hydrogel-based cellular encapsulation. The bicontinuous design of the cryogel skeleton–hydrogel composite yields a material with high permeability to both oxygen and macromolecules, with additional advantages in improved mechanics and cell scaffolding. These properties translate to improved cellular survival and function in low-oxygen cell culture tests. Furthermore, the microscale mechanical interlocking between cryogel skeletons and alginate hydrogels imparts substantial improvements in strength and fracture toughness, which markedly reduces device fracturing in vivo. To ease the immunological response to cell-laden implants, we also integrate an anti-inflammatory polyelectrolyte coating. Together, the composite encapsulation system significantly enhances the survival and function of xenogeneic therapeutic-secreting cells for up to 8 weeks in immunocompetent mice.

## RESULTS

### Development of silicone cryogel skeletons

**Fig. 1.**
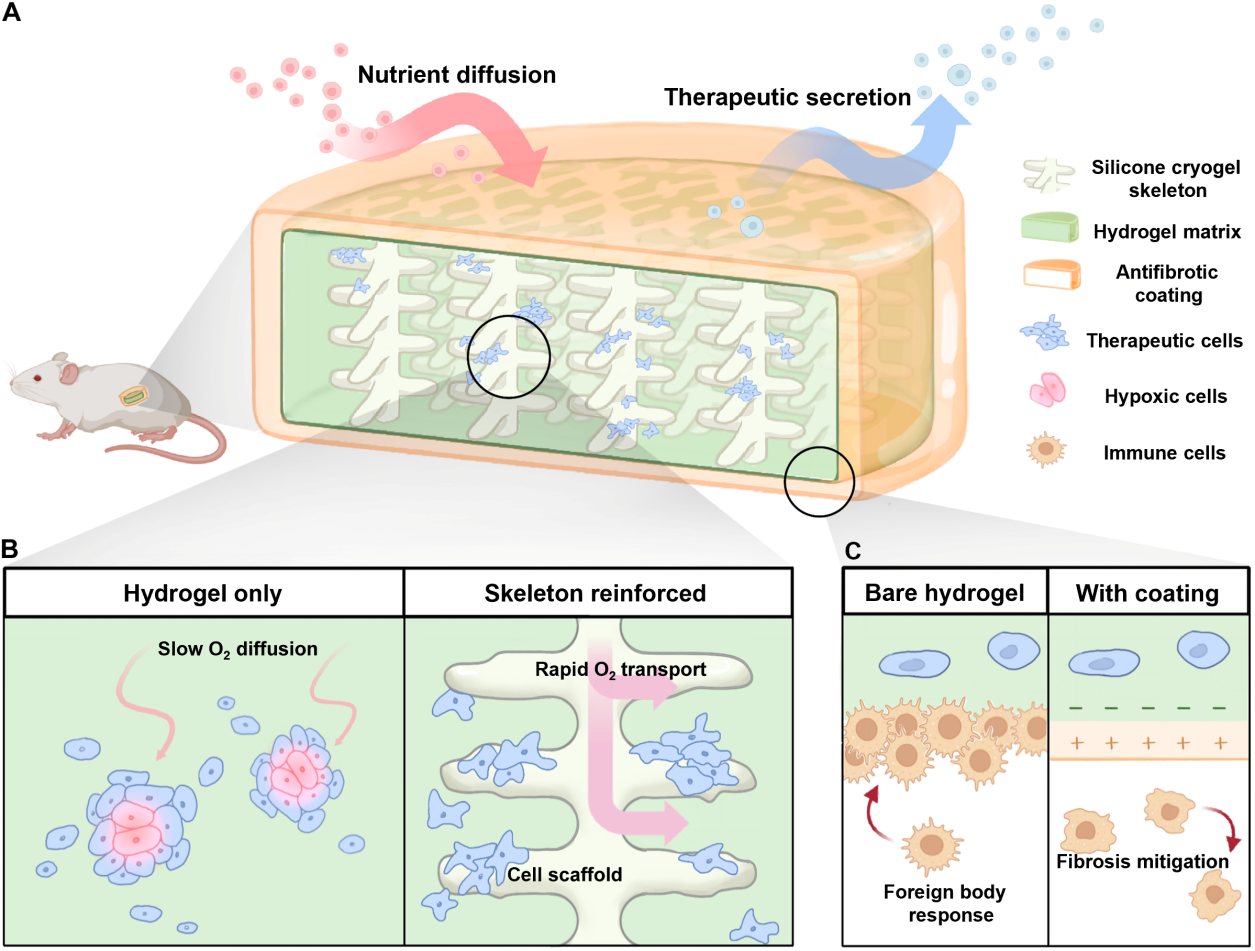
Schematic overview of the composite cell encapsulation system. (**A**) Cross-section view outlining the individual components and the working principle behind the therapeutic implant. Illustrations highlighting the primary functions of the (**B**) silicone cryogel skeleton and (**C**) the antifibrotic coating.

Here, we develop a composite encapsulation system comprising an internal silicone cryogel skeleton, a surrounding hydrogel matrix, and an anti-inflammatory polymer coating (Fig. 1A). The silicone skeleton provides mechanical reinforcement to the fragile hydrogel matrix, facilitates rapid oxygen transport throughout the bulk of the material, and acts as a scaffold for encapsulated cells to grow along (Fig. 1B). Mechanical reinforcement mitigates fracture of implants during handling, implantation, and retrieval, as well as under stresses experienced within the implant niche. The improved oxygen transport and cell scaffolding have important functions in supporting the survival and bioactivity of high densities of therapeutic cells in the absence of implant vascularization. The hydrogel phase supports efficient mass transport of water-soluble nutrients and therapeutics between the implanted device and the host. A positively charged anti-inflammatory polymer electrostatically complexes with the negatively charged alginate hydrogel to form a coating that acts as a countermeasure against the FBR mounted against implants in immunocompetent animals (Fig. 1C).

**Fig. 2.**
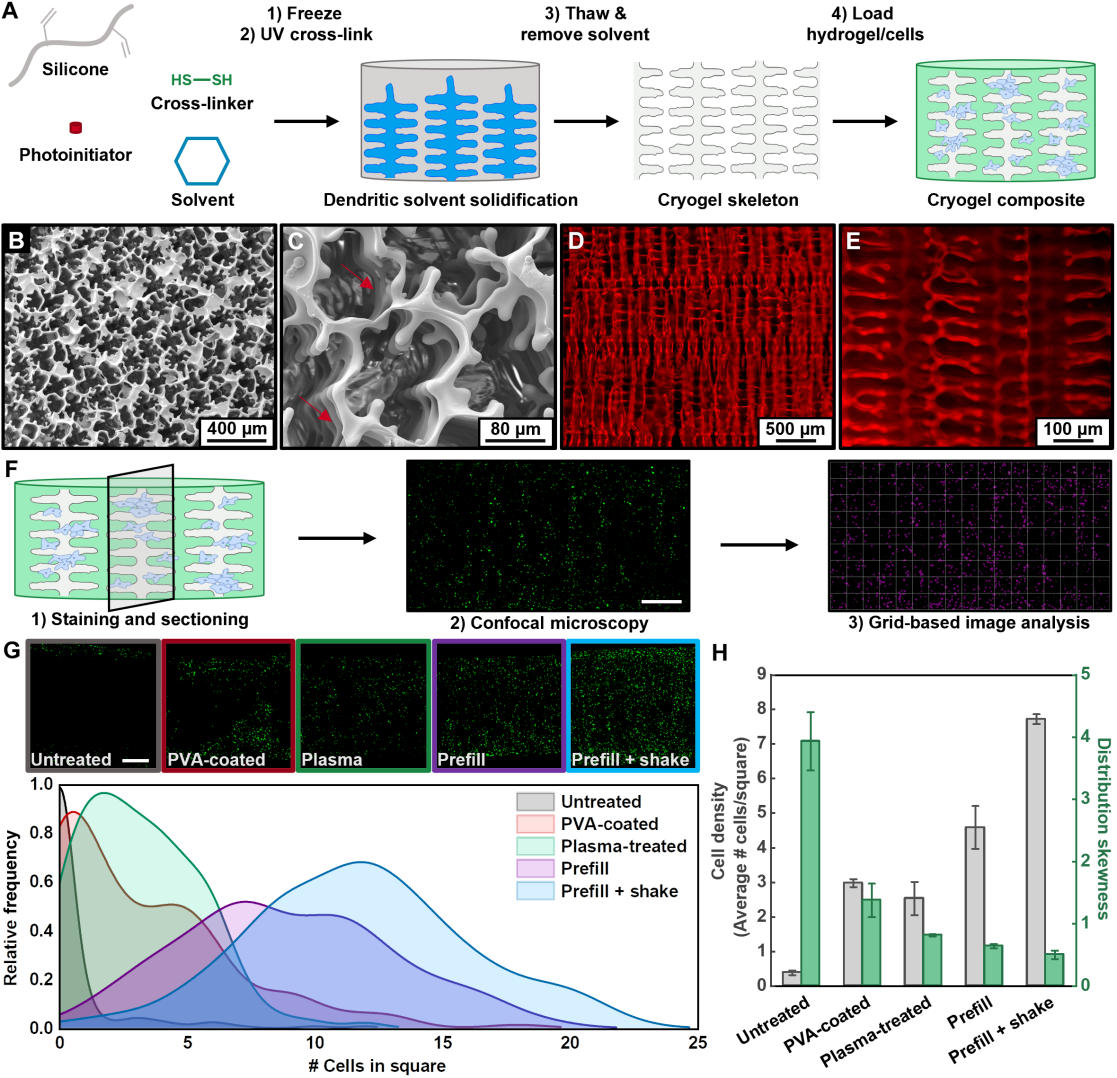
Synthesis and cell loading of silicone cryogel skeletons. (**A**) Scheme illustrating the process for fabricating cell-laden cryogel skeletons. (**B** and **C**) Scanning electron microscopy images of optimized cryogels with clover-shaped surface pores. Arrows indicate transverse connecting pores. (**D** and **E**) Epifluorescence micrographs of cryogels stained with rhodamine B, highlighting the internal dendritic microstructure with long-range straight pathways. (**F**) Overview of the workflow used to characterize the spatial distribution of cells within cryogel skeletons. An image analysis program divides cropped confocal micrographs into a grid of squares and counts the number of cells in each square. Scale bar, 500 μm. (**G**) Exemplary images of samples prepared using various cell loading techniques (top) and the resulting distribution of cell counts after image analysis. Scale bar, 500 μm. (**H**) Plots of the cell density and index of skewness calculated from the distribution data (*n* = 3). Lower skewness indicates a more homogeneous distribution. Error bars represent SE.

Synthesis of silicone cryogel skeletons involves freezing a solution of poly(vinyl-co-dimethyl silicone) oligomers, a dithiol cross-linker, and a photoinitiator dissolved in a solvent, followed by solid-state cross-linking and subsequent thawing and removal of the solvent (Fig. 2A). The solid-state cross-linking process renders a super-macroporous silicone material with the inverse structure of the frozen solvent crystals. Silicones that rely on the classic hydrosilylation reaction typically take several hours at elevated temperatures to cure (*55*). In contrast, the high-speed thiol-ene click reaction achieves rapid (~25 min) cross-linking of the silicone copolymer even under cryogenic conditions. We selected solvents with high melting temperatures such as *tert*-butanol and cyclohexane since they can be conveniently frozen using standard freezers or even in a well-controlled cold room. However, *tert*-butanol tended to produce a fibrous material with anisotropic pores that easily collapse on themselves (fig. S1). In addition, our initial syntheses using cyclohexane following previous reports (*54*) resulted in cryogels that tend to fracture as they de-swell during solvent removal (fig. S2). To generate mechanically resilient cryogels with open pores, we evaluated different polymer loadings and cross-linking densities in cyclohexane. Increasing the cross-linked fraction of available vinyl groups on the silicone generally yields greater compressive moduli (table S1). However, the results indicate that maintaining a low silicone concentration of around 5 wt % in the reaction mix is the most important variable for achieving intact, resilient cryogels (figs. S3 and S4). A wide range of geometries can be achieved by casting bulk cryogel sheets to varying thicknesses and cutting them into the desired shape (fig. S5). This versatile fabrication approach supports rapid, flexible device design for different implant niches and animal models.

Scanning electron microscopy (SEM) of cryogel samples further reveals that modulating the composition, solvent used, and freezing conditions supports a vast diversity of microstructures (fig. S6 and table S1). We hypothesized that a cryogel skeleton with pores of around 150 μm may provide an appropriate growth environment for cells, as this is comparable to the spacing of capillaries in vascularized tissue (*56*). This size regime minimizes the diffusion distance of oxygen permeating from the skeleton into the pore while providing a large enough gap for loading engineered cells (diameter = ~10^1^ μm). The SEM data in Fig. 2B and fig. S7A depict the surface of optimized cryogels, which feature a high density of uniform clover-shaped pores with an average pore size of 143 ± 32 μm. Pore size analysis of three separately synthesized batches of silicone cryogels highlights the reproducibility of the skeleton microstructure (fig. S7B). The characteristic shape of the pores likely corresponds to the formation of orthogonally grown, higher-order dendrites sprouting from a primary dendrite perpendicular to the surface. Higher magnification micrographs taken at a tilted angle indicate a high degree of interconnectivity of the pores in the lateral direction (Fig. 2C). Epifluorescence microscopy of rhodamine stained cryogels (Fig. 2, D and E) and SEM of the cross section (fig. S8) offer better views of the internal microstructure, confirming the presence of an interconnected three-dimensional (3D) network of orthogonally branched pores with millimeter-scale linear channels. Notably, the aligned, elongated pore geometries seen here contrast with the randomly formed, globular pores in previously reported silicone-based materials generated by leaching sacrificial porogens (*57*–*59*). When the skeleton is filled with a hydrogel cell suspension, we hypothesize that these linear pore systems will ease the homogeneous distribution of cells throughout the skeleton. In addition, they may offer optimal channels for swift diffusion of nutrients and therapeutics by minimizing system tortuosity (*60*). Furthermore, the highly interconnected microstructure of the pore walls ensures continuous paths for rapid oxygen transport, as suggested by the percolation theory of conductivity (*61*).

To evaluate the volume contributed by cryogel skeletons to hydrogel composites, we measured the effective porosity of cryogels using two methods that involve either 2D image analysis or measuring the volume of a non-swelling solvent imbibed within the pores. Both techniques yielded a porosity of more than 90%. Since the vast majority of the skeleton volume is available for occupation by the hydrogel cell suspension, the cell density in the hydrogel phase requires only a modest increase to achieve the same global cell density.

### Optimizing cell loading in superhydrophobic silicone skeletons

Efficient, homogeneous distribution of cell suspensions within porous structures is an important challenge in the field of tissue engineering (*62*, *63*). The filling of cells into silicone skeletons is particularly challenging as they exhibit super hydrophobic properties with a water contact angle (WCA) of around 130° (fig. S7). Hence, we first needed to develop a scheme to infiltrate skeletons with aqueous media. Evaluation of the Young-Laplace equation, which relates pressure and surface tension to the behavior of fluid interfaces, led us to three approaches: increasing the surface energy of the skeleton, decreasing the surface tension of the liquid, and increasing the pressure differential across the pore.

Our initial attempts to increase the surface energy of the skeleton involved applying hydrophilic coatings on the silicone cryogels using 3-aminopropyltriethoxysilane (APTES) and polyvinyl alcohol (PVA). However, these coatings yielded only marginal effects on the hydrophilicity of silicone cryogels, which maintained WCAs in excess of 100° (fig. S9). Plasma treatment was the only effective method for making cryogels capable of spontaneously absorbing water. However, microscopy of plasma-treated samples revealed that a large fraction of pores still contained bubbles (fig. S10).

To further improve liquid infiltration, we used ethanol [~22 dyn/cm at room temperature (RT)], which has a surface tension several folds lower compared to that of water (~72 dyn/cm at RT), to pre-wet the pores. Figure S10 shows that swapping ethanol for water alone can achieve similar results to plasma treatment, while combining plasma treatment with ethanol further reduces the number of bubbles. Increasing the pressure differential by briefly placing partially infiltrated samples under a vacuum for 30 s completely removes bubbles in all cases, except for when no surface tension or contact angle modifications are implemented. The ethanol is then removed through aqueous solvent exchange to Dulbecco’s phosphate-buffered saline (DPBS).

After pre-filling skeletons with aqueous media, we endeavored to optimize the filling of skeletons with hydrogel cell suspensions. Techniques to improve the efficiency of loading cells into porous scaffolds generally incorporate some form of mechanical agitation (*62*–*64*). In addition to simply pipetting cell suspensions onto pre-filled skeletons, we tested using a shaker plate to induce agitation and using a centrifuge to force cells into skeletons. To obtain the final cell density, we first quantified the total cell count using a commercial luminescence-based viability assay, and then normalized the cell population by the volume of the entire composite sample. We benchmarked the efficiency of each method against the theoretical cell density, which was determined by factoring in the dilution of the hydrogel cell suspension density by the pre-filled DPBS volume and accounting for the volume contributed by the cryogel skeleton obtained from porosity measurements. The data in fig. S11 indicate that shaker plate agitation significantly improves the cell loading efficiency, while centrifugation has minimal effects. Increasing the shaking time up to 30 min further improves the loading efficiency, achieving an average loading efficiency of 92%.

Nonuniform spatial distribution of encapsulated cells can lead to local oxygen and nutrient depletion in regions of high cell density. As a complement to the overall cell density measurements, confocal fluorescence micrographs of sample cross sections help to visualize the spatial distribution of different cell loading approaches (Fig. 2, F and G). Automated analysis of these images using a quadrat-based algorithm enables quantification of spatial uniformity. The algorithm splits images into a grid of square quadrats, detects the number of cells in each quadrat, and plots a histogram of the cell counts. Calculating the skewness index for the distribution of cell counts provides a quantitative metric for the spatial uniformity of each sample, with lower skewness being more uniform (*65*). Data summarized in Fig. 2 (G and H) highlight the improvements in both cell density and uniformity achieved by our optimized cell loading protocol.

### Mechanical reinforcement of alginate hydrogels and fracture prevention in vivo

To probe the mechanical enhancement afforded by silicone skeletons, we characterized the mechanics of alginate hydrogels and composites cross-linked with 50 mM calcium (Ca-alginate and Ca-composite). As a control, we also evaluated alginate gels cross-linked with a mixture of 95 mM calcium and 5 mM barium (Ba-alginate), which has been demonstrated to enable long-term implantation and retrieval of macroscopic alginate samples in vivo (*52*).

**Fig. 3.**
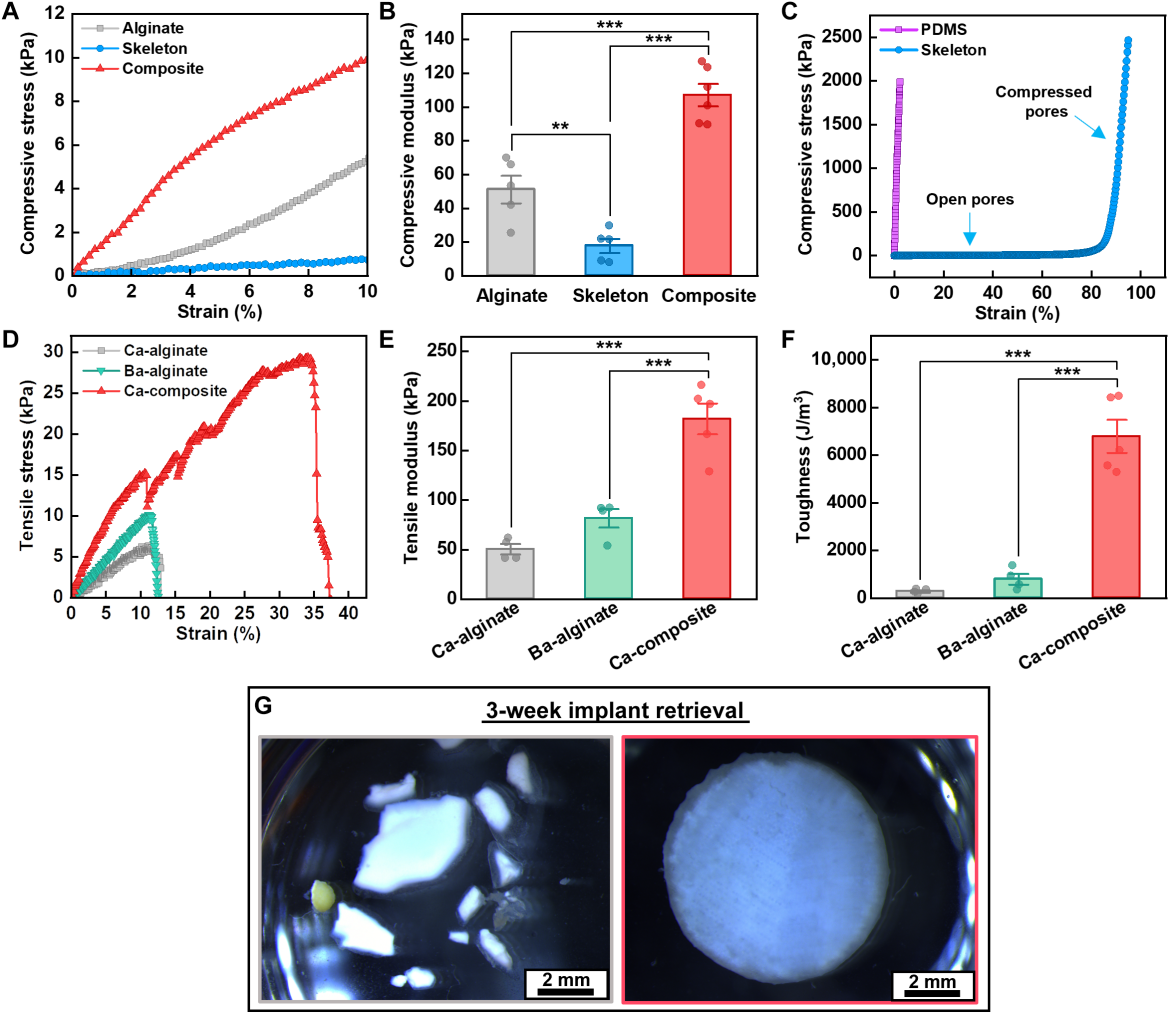
Mechanical reinforcement of alginate hydrogels using cryogel skeletons. (**A**) Stress-strain curves from compression-mode mechanical testing of calcium cross-linked alginate, silicone cryogel skeletons, and calcium cross-linked composites. (**B**) Plot of the compressive moduli of each material. (**C**) Stress-strain curve of polydimethylsiloxane (PDMS) and silicone cryogel skeletons, highlighting the bimodal mechanical properties of skeletons. (**D**) Tensile-mode stress-strain curves comparing the effects of barium cross-linking and skeletal reinforcement. (**E**) Plots of the tensile moduli of each material. (**F**) Plots of the fracture toughness of each material. (**G**) Microscope images of cell-laden samples cross-linked with 50 mM calcium retrieved 3 weeks after implantation in the peritoneal cavity of mice. Alginate samples (left) exhibited substantial fracturing and were retrieved as multiple pieces, while composites (right) remained intact. Error bars for all plots indicate SE. Statistical significance for all plots was determined by one-way analysis of variance (ANOVA) using the Holm-Bonferroni method for multiple comparison correction (**P < 0.01 and ***P < 0.001).

Compression-mode measurements indicate that although cryogel skeletons have a lower compressive modulus than that of Ca-alginate, the composite exhibits a significantly greater compressive modulus compared to either material alone (Fig. 3, A and B). This phenomenon can be explained by examining the compressive-stress strain curve across a larger range of strain (Fig. 3C). Here, it becomes apparent that cryogels exhibit two linear elastic regimes corresponding to when the pores are either open or compressed. In the compressed state, the slope sharply increases and becomes comparable to that of solid polydimethylsiloxane (PDMS) (~10^6^ Pa) which is several orders of magnitude greater compared to most hydrogels (~10^3–4^ Pa). Since this contrast in moduli is comparable to that between epoxy resins (~10^9–10^ Pa) and carbon fibers (~10^11^ Pa) used in high-strength composites, we believe that the observed reinforcement may follow similar mechanisms (*66*, *67*).

Under tensile-mode loading, we see that skeletal reinforcement results in a substantial increase in the ultimate tensile strain and ultimate tensile stress (Fig. 3D). In contrast, chemical reinforcement (Ba-alginate) only provides a modest increase in the ultimate tensile stress. In the linear elastic regime, the average tensile modulus of the Ca-composite is 3.6-fold greater than that of Ca-alginate (Fig. 3E). In addition, the average tensile fracture toughness increases by 23-fold (Fig. 3F), which has important implications on the mechanical resilience of composites. By comparison, barium cross-linking appears to have little effect on fracture toughness.

In composite samples, we consistently noted small dips and recoveries in tensile stress before the maximum strain was reached. However, when we inspected samples in real time, we did not observe any apparent cracks. Given that the first dip occurs near the ultimate tensile strain of alginate gels, it is likely that the dips correspond to internal cracks forming within the hydrogel phase. The skeleton may stabilize these cracks and prevent the overall structure from fracturing. Other studies reporting the formation of internal microcracks within reinforced composites before total failure corroborate this hypothesis (*68*–*70*). These studies report that such internal cracks do not appear to compromise the modulus of composite materials, which is consistent with the stress recovery behavior shown in Fig. 3D. This suggests that even if the internal alginate phase suffers damage, composite implants will remain intact and maintain their mechanical properties.

To evaluate the practical benefits of skeletal reinforcement, disks of Ca-alginate hydrogels (*n* = 4) and Ca-alginate composites (*n* = 3) encapsulating INS-1 rat insulinoma cells were implanted in the intraperitoneal (ip) cavity of BALB/c mice and retrieved 3 weeks later. During retrieval, Ca-alginate hydrogels were found as multiple pieces, often scattered in different locations, indicating that substantial fracturing occurs under stresses experienced in the intraperitoneal space (Fig. 3G). In contrast, all composite samples were found completely intact and easily retrieved. Together, these data suggest that skeletal reinforcement of hydrogel encapsulations significantly reduces mechanical failure, without chemical modifications, and enables facile retrieval of macroscale devices.

### Measurement of oxygen and macromolecular diffusion.

These cryogel skeleton/hydrogel constructs were designed to enhance oxygenation and mechanical strength without negatively affecting the macromolecular diffusion of nutrients and therapeutics. Figure S12 illustrates the experimental setups used for measuring the transport kinetics of both oxygen and fluorescein isothiocyanate–labeled dextran [molecular weight (MW) = 4 kDa] (FITC-dextran) as a surrogate for a small therapeutic macromolecule. In both cases, the diffusivity for a given sample results from tracking the dynamic change of analyte concentration in the acceptor chamber (left) of a side-by-side diffusion cell and calculating the diffusion coefficient using Fick’s law.

**Fig. 4.**
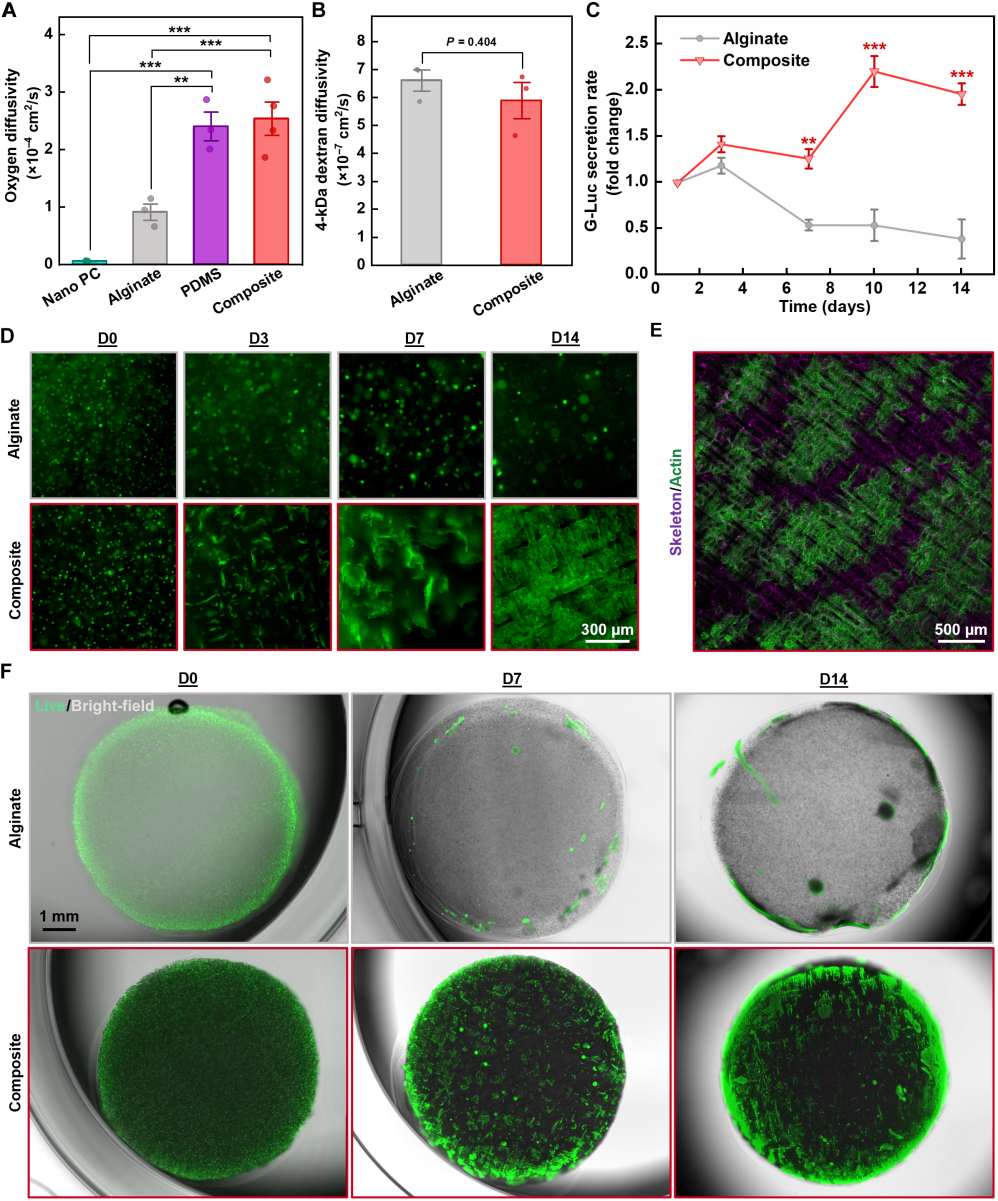
Diffusion measurements and low-oxygen culture experiments. (**A**) Plot of the oxygen diffusivity of a commercial track-etched polycarbonate membrane with 800-nm pores (Nano PC), alginate hydrogel, PDMS, and the alginate-skeleton composite. Statistical significance was determined by one-way ANOVA using the Holm-Bonferroni method for multiple comparison correction (**P < 0.01 and ***P < 0.001). (**B**) Comparison of the diffusivity of fluorescently labeled 4-kDa dextran in alginate and the composite. The *P* value presented was calculated from an unpaired, two-tailed *t* test. (**C**) Gaussia luciferase (G-Luc) secretion rates measured from samples containing HEK-GLuc cells cultured at 5% oxygen over 14 days (*n* = 3 alginate, *n* = 4 cryogel composite). Statistical significance for the comparison of cryogel composite and alginate groups at each time point was determined from unpaired, two-tailed *t* tests (**P < 0.01 and ***P < 0.001). (**D**) Time-sequenced epifluorescence micrographs of actin-stained (green) HEK-293T cells revealing differences in morphological development when encapsulated in either alginate or composite. Samples were cultured at 5% oxygen and fixed at various time intervals before staining and imaging. (**E**) *Z*-Stack confocal image of stained HEK-293T cells that were cultured for 14 days in a composite with fluorescent quantum dots dispersed within the skeleton. (**F**) Overlaid fluorescence and bright-field micrographs of live HEK-GLuc cells encapsulated in either alginate or skeletal composite and stained using calcein AM viability dye (green) at various time points. Error bars for all plots indicate SE.

The results summarized in Fig. 4 (A and B) show that skeletal reinforcement of alginate imparts a 2.8-fold increase in oxygen diffusivity while negligibly affecting the macromolecular diffusivity of FITC-dextran. Commercial nanoporous polycarbonate membranes (Nano PC) used for immunoisolation in previous macroscale cell encapsulation platforms (*29*) exhibit at least an order of magnitude lower oxygen diffusivity compared to all other materials tested, highlighting the potential benefits of membrane-free devices.

### Evaluation of cellular function under low-oxygen culture conditions

We performed in vitro cell culture experiments to investigate the cellular function of silicone skeletons integrated with alginate cell suspensions. To simulate the environment experienced by implants in the peritoneal cavity, we used an incubator with the oxygen partial pressure controlled at 5% (*9*). Samples consisted of disks of alginate (*n* = 3) and alginate-skeleton composites (*n* = 4) containing human embryonic kidney (HEK) cells engineered to secrete Gaussia luciferase (G-Luc) (HEK-GLuc).

Monitoring the change in G-Luc secretion rate over 14 days provides insights into the survival and function of encapsulated cells (Fig. 4C). The data indicate an increasing secretion rate in skeletal composites and a diminishing secretion rate in alginate samples, with the disparity between the two groups becoming significant by day 7 (**P < 0.01). Although both groups exhibit a slight increase in secretion rate over the first 3 days, alginate samples exhibit a sharp decrease over the following 4 days (**P < 0.01) while cryogel samples remain at similar levels (*P* = 0.3837). Over the course of the second week, secretion from skeletal composites rises to about double the initial secretion rate, while alginate samples remain at around half their original secretion rate.

In a repeat experiment using unmodified HEK-293T cells, samples that were fixed and fluorescently stained at various time intervals revealed pronounced differences in cell morphology (Fig. 4D). Over the course of the study, cells in alginate samples remained as spherical clusters with minimal enlargement of the cluster diameter. In contrast, cellular growths within skeletal composites exhibited significant symmetry breaking by day 3 and continued to spread into larger formations through day 14. Prior studies on tissue development report that such symmetry-breaking events directly correlate with higher rates of proliferation and are strongly influenced by the mechanical environment of the cells (*71*, *72*). Overlaying polarized bright-field images (fig. S13) led us to believe that proliferating cells tend to grow along the surface of the skeletons. To confirm this, we repeated the experiment with composites formed using skeletons doped with fluorescent quantum dots. Confocal laser scanning microscopy of day 14 samples shows clear colocalization of the fluorescent skeleton and the actin-stained cells (Fig. 4E).

Imaging of live samples stained at various time points with a fluorescent viability dye (Fig. 4F), combined with measurements of total extractable DNA on day 14 (fig. S14), provide additional, direct evidence of improved cell viability and proliferation when encapsulated in skeletal composites. We observed that in both encapsulation materials, viability was greatest along the periphery, likely due to improved access to nutrients and reduced competition from neighboring cells. Movie S1 showcases a 3D reconstruction of these live-stained cells proliferating along a quantum dot-laden skeleton after 2 weeks of culture.

These hypoxic cell culture studies demonstrate that skeletal reinforced composites can enable higher levels of protein secretion from engineered therapeutic cells at physiologically relevant oxygen concentrations. Furthermore, a unique cell scaffolding effect leads to potentially favorable cellular morphology and opens avenues for additional functionality through chemical modification of the silicone surface with biochemical signaling cues.

### Mitigating fibrosis using poly(β-amino alcohol) coatings

**Fig. 5.**
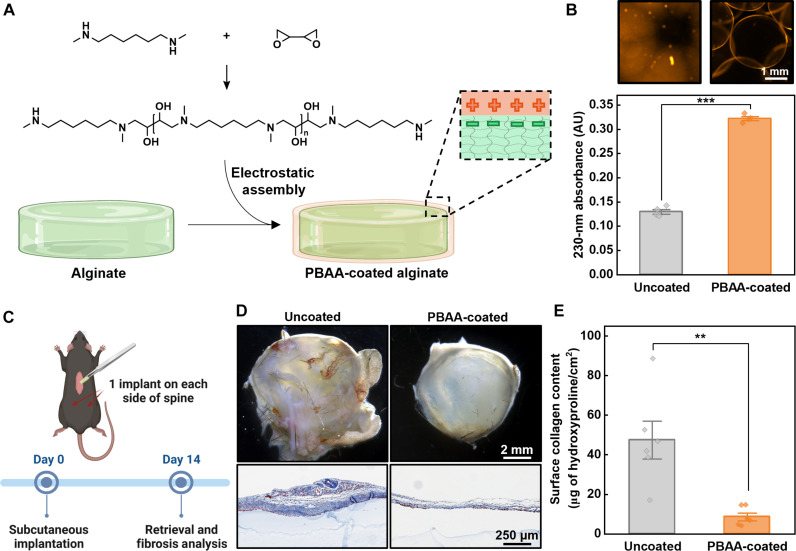
PBAA coating to reduce fibrosis. (**A**) Schematic of the chemical synthesis and assembly mechanism of the PBAA coating. (**B**) Comparison of the 230-nm light absorbance of alginate samples before and after coating with PBAA polymer. Statistical significance was determined by an unpaired, two-tailed *t* test (***P < 0.001). Phase contrast micrographs above the plot illustrate the formation of a secondary phase localized around the periphery of alginate microparticles. (**C**) Schematic outlining in vivo experiment for evaluating the antifibrotic performance of PBAA polymer coatings. Uncoated and coated groups each involved three mice with two implants per mouse. (**D**) Microscope images (top) and histological cross sections with Masson’s trichrome stain (bottom) of explanted alginate hydrogels with and without the PBAA coating. (**E**) Biochemical quantification of surface collagen on implant surfaces by hydroxyproline assay. Statistical significance was determined by an unpaired, two-tailed *t* test (**P < 0.01).

In preliminary in vivo trials, we found the buildup of fibrotic tissue on the surface of implants negatively affected the longevity of encapsulated cells. This issue is frequently cited as a critical hurdle barring the long-term function of other cell encapsulation platforms (*29*, *39*, *48*, *73*). As a countermeasure, a cationic poly(β-amino alcohol) (PBAA) was used as a coating, as it was previously demonstrated to reduce fibrosis (Fig. 5A) (*74*). The synthesis of PBAA uses an epoxide aminolysis reaction, which produces a polymer with an MW of around 3 kDa as measured by nuclear magnetic resonance (NMR) end group analysis (3222 Da) (fig. S15A) and matrix-assisted laser desorption/ionization–time-of-flight (MALDI-TOF) mass spectrometry (3111 Da) (fig. S15B). The ultraviolet-visible (UV-vis) spectra of PBAAs indicate a sharp absorption peak at 230 nm, with absorption increasing linearly with concentration (fig. S16, A and B). Alginate microcapsules treated with PBAA and thoroughly washed with distilled water show a significant increase in absorption at 230 nm indicating successful complexation (Fig. 5B). Phase contrast microscopy of capsules before and after coating indicates the generation of a secondary phase on the surface of each capsule (Fig. 5B).

To test the antifibrotic effects of the PBAA coating on macroscale hydrogel implants, we subcutaneously implanted alginate disks (diameter = 7 mm, thickness = 2 mm) with or without PBAA coating onto the backs of C57BL/6 mice for 14 days (Fig. 5C). Upon retrieval, PBAA-coated implants appeared noticeably less fibrosis and exhibited significantly thinner capsule thickness as revealed by Masson’s trichrome staining of explant cross sections (Fig. 5D and fig. S17). These results are corroborated by biochemical quantification of tissue harvested from the explants, which indicates a significant reduction in surface collagen (Fig. 5E). Immunohistochemistry (IHC) performed using antibodies specific for activated myofibroblasts (α–smooth muscle actin) and macrophage (F4/80) provides additional insights into the composition of the cellular overgrowth (fig. S18). IHC-stained histological sections showcase a characteristic layer of myofibroblasts lining the interface of the fibrotic tissue and the implant. Both the thickness of this layer and the density of macrophages appear to be substantially reduced in PBAA-coated samples.

For coating functional implants containing protein-secreting cells, we applied a dipcoat of acellular alginate to provide a pristine surface for PBAA complexation (fig. S19A). The PBAA coating proves to be well tolerated by encapsulated cells, as evidenced by viability measurements of encapsulated HEK-GLuc cells before and after complexation (fig. S19B). G-Luc secretion measurements taken over 3 days show that both the alginate dipcoat and the PBAA coating negligibly affect protein secretion, suggesting that functionality perseveres following coating (fig. S19C).

### Enhanced therapeutic implants using cryogel skeletal composites in vivo

**Fig. 6.**
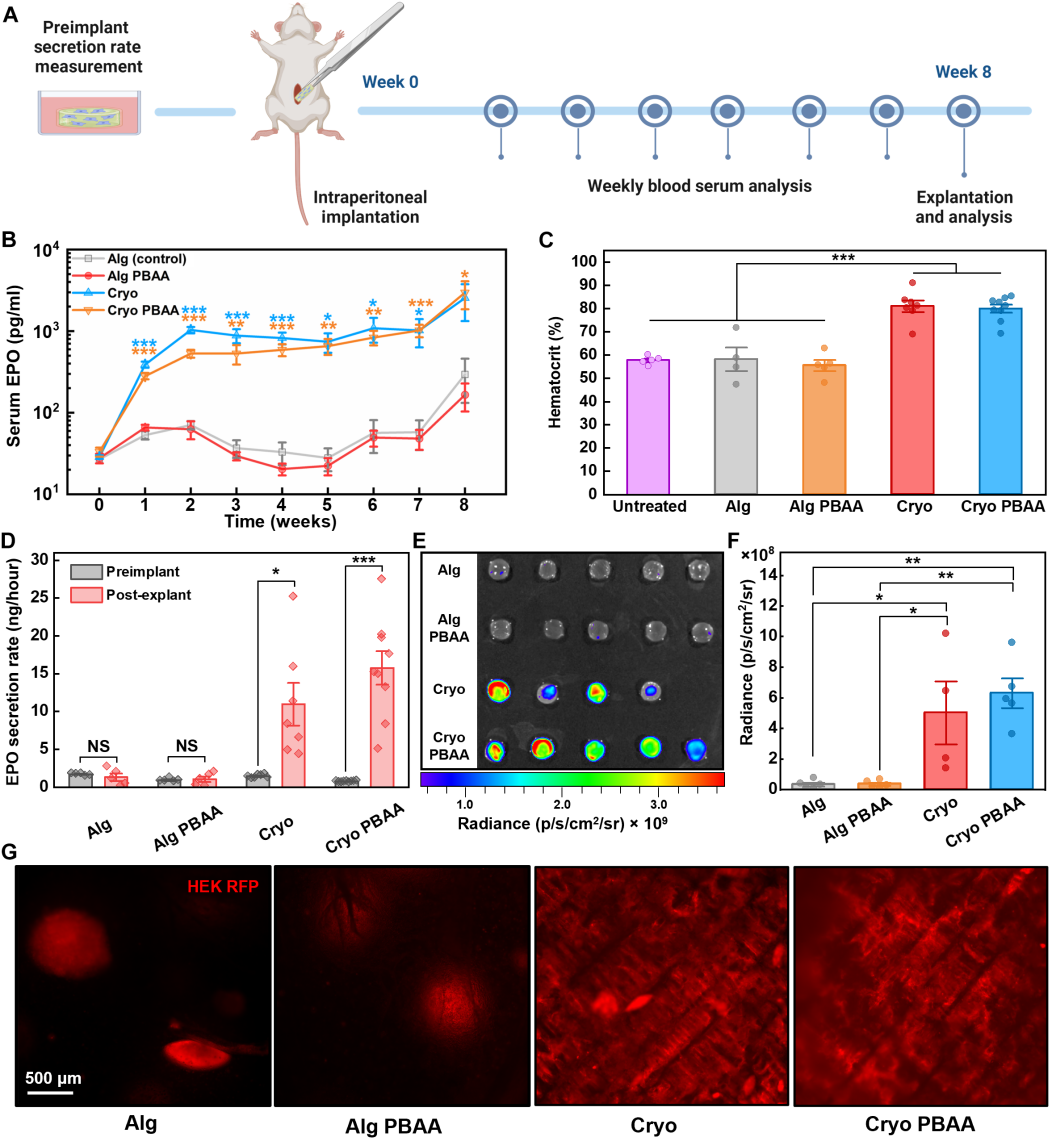
Enhanced long-term delivery of therapeutics in BALB/c mice from HEK-EPO cells encapsulated in cryogel composites. (**A**) Schematic overview of the mouse study. (**B**) Plot of EPO levels in mouse serum over 8 weeks. Week 0 time points represent basal serum EPO levels in mice with no implants. Alg, alginate (*n* = 5); Alg PBAA, PBAA-coated alginate (*n* = 5); Cryo, silicone cryogel composite (*n* = 7); Cryo PBAA, PBAA-coated composite (*n* = 9). Statistical significance for each time point was determined by an unpaired, two-tailed *t* test comparing each test group against the Alg group. Asterisk colors correspond with the colors of the significant test groups. The data represents studies performed across two separate cohorts of animals. (**C**) Plot of hematocrit measurements taken after 8 weeks of treatment. Significance was computed by one-way ANOVA using the Holm-Bonferroni method for multiple comparison correction. (**D**) Comparison of EPO secretion rates measured in vitro before and after implantation. Statistical significance was determined from unpaired, two-tail *t* tests. (**E**) Bioluminescence imaging of retrieved implants incubated with luciferin. (**F**) Quantification of bioluminescence. Significance was determined by one-way ANOVA using the Holm-Bonferroni method for multiple comparison correction. (**G**) Epifluorescence micrographs of HEK-EPO cells expressing RFP within retrieved implants, revealing differences in cell morphology as seen in in vitro experiments. For all plots, error bars indicate SE and significance markers follow: *P < 0.05, **P < 0.01, and ***P < 0.001.

To assess the effects of cryogel skeletons and the PBAA coating on encapsulated cell therapies, we engineered a HEK cell line to secrete erythropoietin (EPO), a model therapeutic protein commonly administered to treat anemia (red blood cell deficiency) associated with a variety of diseases including cancer, renal failure, and HIV/AIDS (*75*). These HEK-EPO cells were also modified to express both firefly luciferase (F-Luc) and red fluorescent protein (RFP) to facilitate luminescent and fluorescent evaluation of cell viability and morphology within explanted samples. For in vivo evaluation of implant performance, we encapsulated these cells in different materials before implantation in the peritoneal cavity of BALB/c mice (Fig. 6A).

In the initial phase of our study, we applied a 1 wt % alginate dipcoat to all samples and tracked mice serum EPO levels over 4 weeks (fig. S20A). Skeletal composite encapsulated therapies (Cryo) produced significantly higher EPO levels than their alginate (Alg) counterparts for the first 2 weeks (fig. S20A). However, in the absence of the PBAA coating, these levels rapidly decreased in weeks 3 and 4. The decline seen in Cryo samples could be attributed to both elevated fibrosis (fig. S20B) and compromised alginate dipcoats, as only 10% of these samples retained their outer alginate layer (fig. S21, A and B). The adverse effects of dipcoat damage are illustrated by murine macrophage-specific IHC staining of explants, which reveals extensive immune cell infiltration in uncoated areas (fig. S21C). In contrast, intact coatings robustly isolate the implant. We found that both increasing the alginate dipcoat to 1.4 wt % and applying the PBAA coating proved effective in improving coating integrity (fig. S21B).

Figure 6B summarizes serum EPO levels tracked over 8 weeks from mice treated with samples coated with 1.4% alginate. We observed that both Cryo and Cryo PBAA groups displayed EPO levels nearly an order of magnitude higher than the Alg and Alg PBAA groups after just 1 week of implantation and maintained similarly elevated levels throughout the study. As a metric of therapeutic efficacy, we quantified the hematocrit at week 8 (Fig. 6C), which represents the percentage of blood volume comprised of red blood cells. The measurements indicate that both alginate groups yielded negligible effect compared to untreated mice, while Cryo and Cryo PBAA implants led to an average increase of 21 and 23%, respectively. These significant increases in hematocrit serve as a strong indicator of therapeutic potential considering that anemic mice typically exhibit a drop of around 8 to 17% (*76*, *77*).

A subsequent series of ex vivo measurements sought to examine metabolic and morphological changes in the encapsulated cells over the course of the implantation period. Comparing pre- and postimplantation data showed that average EPO secretion rates from Cryo and Cryo PBAA groups increased by approximately an order of magnitude, while those of Alg and Alg PBAA samples remained relatively unchanged (Fig. 6D). Notably, the Cryo PBAA group achieved the most significant enhancement, with a rise of 20.8-fold. Bioluminescent imaging, facilitated by constitutive F-Luc expression in HEK-EPO cells, enabled the quantification of viable cell populations in samples incubated with luciferin (Fig. 6, E and F). These viability results echo the trends observed in EPO and hematocrit measurements. In addition, live fluorescence imaging of the RFP-expressing cells post-explantation showed that transplanted cells recapitulate the morphological differences seen in vitro (Fig. 6G). Specifically, cell clusters in both skeletal composite groups exhibit distinct symmetry disruptions and apparent growth along the skeleton.

Histological analysis and hydroxyproline quantification again indicated that composite samples without PBAA exhibited the highest degree of fibrosis (fig. S22, A to D). Likely, this is due to the larger population of xenogeneic HEK cells, whose secreted antigens may elicit a more pronounced immune reaction compared to allogeneic or autologous analogs. Combined with the EPO level measurements, these findings suggest that the reduced function in 1% alginate-coated Cryo samples (fig. S21A) was predominantly due to immune cell attack rather than impaired transport due to fibrosis. However, we expect that the significant reduction in fibrosis conferred by the PBAA on skeletal composites will yield greater utility in higher-order animals, including humans, where the FBR is typically more vigorous (*39*).

## DISCUSSION

The soft composite encapsulation system reported here offers a promising approach to improving the performance and practicality of cell-based therapeutic devices. Our results demonstrate that silicone cryogel skeletons significantly boost oxygen transport in alginate hydrogels, without compromising macromolecular diffusivity, and act as physical scaffolds for supporting 3D cellular proliferation. These functionalities support higher densities of encapsulated cells, facilitating more compact therapeutic implants that are easier to implant and less invasive. We also show that these skeletons substantially improve the mechanical properties of alginate hydrogels, opening avenues for macroscale alginate implants without the use of toxic barium or external housings that impede diffusion. Moreover, the development of an anti-inflammatory PBAA coating further enhances the system by reducing fibrotic encapsulation and extending the performance advantages of skeletons in vivo.

By decoupling device mechanics from the hydrogel chemistry, cryogel skeletons open opportunities for modifying the hydrogel phase with bioactive peptides or extracellular matrix components to enhance the potency of encapsulated cell therapies. Furthermore, a broad range of commercial silane coupling reagents could support additional functionalization of the cryogel skeletons such as engrafting cell-signaling moieties.

The performance benefits of the cryogel skeleton are partially attributed to its unique microstructure, which results from dendritic solidification during synthesis. Its high porosity (>90%) enables dense cell loading with minimal increase in volume. The 3D orthogonal pore system is characterized by high interconnectivity and low tortuosity, with linear pores that extend over centimeters to enable both homogeneous cell filling and efficient diffusion of nutrients. These features represent distinct advantages over conventional porogen-leached silicone materials (*57*, *58*). The random organization of porogens results in high tortuosity and may leave inaccessible voids if the sacrificial particles are not in contact with each other. For example, a prior study on salt-leached silicone sponges, fabricated using 90% v/v NaCl/silicone, found that particles sized 150 to 250 μm yielded an effective porosity of 85% and interconnectivity of 67%, while particles sized 53 to 106 μm yielded a further reduced effective porosity of 73% and interconnectivity of only 37% (*59*). Furthermore, the authors observed that the pores in these structures collapsed when the porogen volume fraction exceeded 90%.

While cryogels have been demonstrated in a variety of biological engineering applications, these materials are mostly synthesized using water-soluble biopolymers such as gelatin and collagen (*78*). Previous attempts at using such materials for therapeutic cell encapsulation revealed that encapsulated cells are highly susceptible to hypoxia without the aid of oxygen-generating particles (*46*), which deplete over time. Furthermore, little work has been done to reduce the fibrotic response to such cryogel systems. By contrast, studies on cryogels formed from hydrophobic elastomers and plastics have previously been confined to environmental applications such as the recovery and remediation of oil spills (*54*, *79*, *80*). As such, we developed a hybrid hydrophobic cryogel-hydrogel composite specifically designed to improve the oxygenation and long-term function of encapsulated cell therapies.

A key aspect of the presented platform is its potential to address a broad spectrum of diseases requiring protein replacement by modifying the transgenic cell line to secrete alternative biologics. Furthermore, although we tailored the skeletons for use with engineered cell suspensions, optimization of the composition and synthesis conditions to create larger pore sizes could facilitate compatibility with islets for treating type 1 diabetes. We also anticipate that the individual components of the current system may find utility in adjacent fields. For instance, the skeleton might be integrated with other hydrogels for tissue engineering applications that demand a 3D scaffold or involve high cell densities or hypoxic environments. In addition, the anti-inflammatory attributes of the PBAA coating may be harnessed to minimize fibrosis on medical sensors or serve as a localized materials-based immunosuppressant. Together, the materials and design principles presented herein collectively have the potential to bolster the effectiveness of cell-based therapies and contribute to advancements in diverse biomedical disciplines.

## MATERIALS AND METHODS

### Materials

All chemicals were purchased from Sigma-Aldrich and all cell culture reagents were purchased from Thermo Fisher Scientific unless otherwise stated. Seven to 8% poly(vinylmethylsiloxane-co-dimethylsiloxane) (PVDMS) and trichloro(1*H*,1*H*,2*H*,2*H*-perfluorooctyl)silane were purchased from Gelest (product code: VDT-731). The cross-linker, 1,6-hexanedithiol (W349518; Sigma-Aldrich), and photoinitiator, 2,2-dimethoxy-2-phenylacetophenone (DMPA) (catalog no. 196118) were purchased from Sigma-Aldrich. 1,3-Butadiene diepoxide and *N*,*N*′-dimethyl-1,6-hexanediamine for the PBAA synthesis were purchased from Sigma-Aldirch. PRONOVA SLG20 sodium alginate was purchased from NovaMatrix. PVA was procured from Polysciences Inc. (catalog no. 02975). SYLGARD 184 silicone elastomer kit was obtained from Ellsworth Adhesives. Nanoporous polycarbonate membranes were purchased from Sterlitech. F-Actin–specific Alexa Fluor 488–conjugated phalloidin was purchased from Life Technologies. Gaussia Luciferase assay kits were purchased from Thermo Fisher Scientific (catalog no. 16160). The CellTiter-Glo 3D Luminescent Cell Viability Assay was purchased from Promega (catalog no. G9681). The DNA extraction kit was obtained from Qiagen (catalog no. 69504). The hydroxyproline assays used for collagen measurement were obtained from either Sigma-Aldrich (catalog no. MAK008) or Chondrex (catalog no. 6017). Mouse EPO enzyme-linked immunosorbent assay (ELISA) kits were purchased from BioLegend (catalog no. 442780). HEK-293T cells were purchased from the American Type Culture Collection. Anti-mouse antibodies for α–smooth muscle actin and F4/80 were obtained from Sigma-Aldrich (catalog no. ABT1492) and Cell Signaling Technologies (catalog no. 70076), respectively.

### Microscopy

SEM was performed using a ZEISS Crossbeam 540 field emission scanning electron microscope. Samples were affixed to SEM stubs using conductive carbon tape and conductive silver paint around the periphery of the sample. Samples were then sputtered with a thin layer of gold. Epifluorescence and phase contrast microscopy of skeleton microstructure and PBAA coatings respectively used a Nikon ECLIPSE Ts2 inverted microscope, while epifluorescence and polarized bright-field images of cell morphology were taken on a DeltaVision Ultra Microscope. Paneled images of whole samples for cell viability evaluation were captured using a Keyence BZ-X810 fluorescence microscope and stitched using the integrated image processing software. Bright-field images of the fluid infiltration studies and retrieved implants were recorded using a Leica stereoscopic microscope. Confocal micrographs were taken on an Olympus FV1200 laser scanning confocal microscope. Histological slides were scanned using an Aperio slide scanner.

### Synthesis of silicone cryogel skeletons

The synthesis of silicone cryogels followed a modified version of a method previously reported by Ozmen *et al*. (*54*). The optimized reaction mixture comprised 5% w/v PVDMS prepolymer, 0.47% w/v 1,6-hexanedithiol cross-linker, and 0.05% w/v DMPA vigorously mixed in cyclohexane (Sigma-Aldrich) and allowed to equilibrate for at least 15 min protected from light. In the synthesis of fluorescently doped cryogels used in Fig. 4E, a colloid (1 mg/ml) of hydrophobic CdSeS/ZnS quantum dots in toluene (Sigma-Aldrich, catalog no. 753807) was added to the reaction mixture at a volume ratio of one part colloid to six parts PVDMS. An appropriate volume of the reaction mix was then transferred to the reaction vessel and placed on a metal wire rack in a freezer held at −12°C before sealing the vessel. The mix was frozen for 75 min before the vessel was immediately placed in a Styrofoam box containing ice and transferred to a cold room (4°C). Uncovering the vessel and irradiating the frozen reaction mix on ice with ultraviolet light for 25 min facilitated solid-state cross-linking of the PVDMS. The resulting cryogel was then washed in sequential baths of hexane (overnight), ethyl acetate (1 hour), and isopropyl alcohol (1 hour) to remove unreacted reagents while enabling gradual deswelling of the cryogel. A metal spatula was used to gently release the cryogel from the glass walls and transfer it onto a nonwoven wiper. Last, the cryogel was lightly compressed to remove most of the entrapped alcohol before placing it in a vacuum desiccator to dry overnight.

For the composition screen, syntheses took place in 20-ml glass vials (VWR). In all other experiments, cryogels were synthesized in a 100-mm diameter glass petri dish (Corning). To enable the facile release of cryogels, dishes underwent a fluorination treatment. Briefly, dishes were placed in a vacuum desiccator along with an uncapped 1.5-ml microtube containing 20 μl of trichloro(1*H*,1*H*,2*H*,2*H*-perfluorooctyl)silane overnight. Before each synthesis, reused petri dishes were cleaned vigorously with deionized water and detergent (Alconox) followed by drying under a stream of nitrogen gas and dehydration in an oven at 120°C.

### Fabrication of cryogel-hydrogel composites

Dried cryogels were first cut into desired shapes using either scissors for tensile mechanics and diffusion test samples or biopsy punches for compression test samples and cell-containing samples. Cryogels were then hydrophilized by air plasma treatment (Harrick Plasma Cleaner; 500 to 700 mtorr; high radio frequency) for 90 s on both sides. Hydrophilized cryogels were then transferred into a vial containing ethanol and sealed with a screw cap containing a septum to both pre-wet and sterilize the cryogels. The remaining air bubbles were evacuated by exposing the airspace above the ethanol to vacuum by piercing the septum with a 16-gauge needle connected to a vacuum line for 30 s. Samples were left to sterilize in the ethanol for at least 15 min before undergoing an aqueous solvent exchange process whereby the alcohol-imbibed cryogels were sequentially soaked in a sterile water bath followed by sterile DPBS with gentle agitation.

Acellular composites were formed by immersing pre-filled cryogels in a solution of 1.4% SLG20 alginate dissolved in sterile saline and placed on a shaker plate for at least 30 min at 500 RPM. Samples were then placed on a polystyrene petri dish and excess alginate solution was dabbed away. Cryogel-hydrogel composites were lastly formed by cross-linking in a bath of either 50 mM CaCl_2_ (Ca-gelling solution) or 95 mM CaCl_2_ + 5 mM BaCl_2_ (Ba-gelling solution) for 30 min. Composites were thoroughly rinsed with deionized water to remove excess ions.

Composite encapsulation of cells began by suspending cells in a 1.4% solution of SLG20 in sterile saline. The suspension was pipetted on top of pre-filled cryogels (diameter = 7 mm, thickness = 2 mm) in a tissue culture well plate and then agitated for 30 min on a shaker plate at 500 RPM. Samples were flipped midway during the agitation process using sterile forceps. Cross-linking of cell-laden samples occurred in either the Ca-gelling solution or the Ba-gelling solution supplemented with 15 mM Hepes for 30 min. Composite samples were then soaked in 300 mM d-mannitol and 50 mM Hepes to remove excess ions followed by brief rinsing with DPBS before incubation in cell culture media. Samples designated for in vivo experiments were dip-coated twice with either a 1 or 1.4% solution of SLG20 alginate and cross-linked to form an acellular layer of alginate (~100 μm thickness), which helped to prevent cellular escapement and ensured a continuous alginate surface for coating with PBAA.

### Hydrophilic coatings on silicone skeletons

Application of hydrophilic coatings on cryogel skeletons began by plasma treating dry cryogels for 90 s on both sides to activate the surface of the silicone. PVA coating involved both sides before immediately immersing skeletons in a 1 wt % aqueous solution of PVA (~25,000 Da, 88 mol % hydrolyzed) for 20 min. Skeletons were then removed and compressed with delicate task wipes to remove excess fluid before baking for 15 min in a 120°C oven. APTES-coated skeletons were achieved by placing plasma-treated skeletons in an ethanolic solution containing 2% APTES and 3% water. Vacuum was then applied following the procedure described in the fabrication of cryogel-hydrogel composites to remove residual bubbles. Samples were allowed to react overnight at RT before washing in ethanol three times, for 10 min each. Coated samples were then compressed with delicate task wipers to remove excess fluid and baked at 120°C for 1 hour.

### Fabrication of alginate hydrogels

Acellular alginate hydrogels were fabricated by injecting the 1.4% SLG20 alginate solution into a plastic mold with the desired thickness covered with a semiporous membrane. Molds were then immersed in either the Ca-gelling solution or the Ba-gelling solution for 1 hour before removing the cross-linked gel and soaking in deionized water to remove excess ions.

Alginate cell suspensions were supplemented with an iodixanol-based density gradient medium (OptiPrep) to achieve a final concentration of 8% iodixanol, which we experimentally found to prevent the gravitational settling of cells during gelation. Cross-linking occurred in either the Ca-gelling solution or the Ba-gelling solution supplemented with 15 mM Hepes for 1 hour before removing gels and punching out disks (diameter = 7 mm, thickness = 2 mm) using a biopsy punch. Samples were then soaked in 250 mM d-mannitol and 50 mM Hepes to remove excess ions followed by brief rinsing with DPBS before incubation in cell culture media. Samples designated for in vivo experiments were dip-coated twice with a 1% solution of SLG20 alginate and cross-linked to form an acellular layer of alginate (~100 μm thickness), which helped to prevent cellular escapement and ensured a continuous alginate surface for coating with PBAA.

### Preparation of fluorescently stained samples

Rhodamine-stained samples resulted from soaking cryogels in a solution (1 mg/ml) of rhodamine B overnight before rinsing with deionized water.

Actin-stained samples involved in cellular distribution and cell morphology studies were first fixed with 10% formalin for 45 min. Fixed samples were then rinsed briefly with DPBS before soaking in DPBS for several hours. After fixation and rinsing, samples were permeabilized for 45 min in 0.1% Tween 20 in DPBS containing calcium and magnesium. After rinsing twice with DPBS, permeabilized samples were stained with F-actin–specific Alexa Fluor 488–conjugated phalloidin. Last, samples were cleared using a 70% glycerol solution.

For fluorescence-based visualization of cell viability, samples were incubated with 2 mM Calcein AM in media for 30 min at 37°C. Samples were then rinsed with DPBS before imaging.

### Preparation of PDMS membranes

PDMS membranes for diffusion studies were prepared using SYLGARD 184 silicone elastomer kits with a base–to–curing agent ratio of 10:1. Samples were cast in a polystyrene petri dish and cured in a 60°C oven.

### Synthesis and characterization of PBAA

The polymerizations took place neatly in bulk at RT under an inert atmosphere with 1,3-butadiene diepoxide (1 equivalent) and *N*,*N*′-dimethyl-1,6-hexanediamine (1.2 equivalents) under vigorous stirring for 18 hours. ^1^H NMR spectroscopy (Bruker Advance 500, 500.18 MHz) of samples prepared in deuterium oxide confirmed the successful synthesis of the polymer (fig. S15A). End group analysis comparing the proton on secondary amines (δ 2.14 to δ 2.20) to the methyl proton on tertiary amines (δ 3.14 to δ 3.17) resulted in an average MW of 3222 Da. Linear mode MALDI-TOF mass spectrometry (Bruker microflex) using an α-cyano-4-hydroxycinnamic acid matrix yielded a similar average MW of 3111 Da. A microplate reader (Tecan) facilitated UV-VIS spectroscopy of samples prepared in a UV-transparent 96-well plate (Greiner Bio-One).

### PBAA coating

In preparation for coating, the PBAA was first dissolved in a mannitol buffer containing 300 mM d-mannitol and 25 mM Hepes to a concentration of 0.3 wt %, and the pH was adjusted to around 7.2 using 6 M HCl. The final coating solution was diluted to a final concentration of 0.01 wt % with the mannitol buffer before sterile filtering.

PBAA coating immediately followed the preparation of gelled samples. Samples were first incubated for 5 min in a Hepes buffer [25 mM Hepes, 1.2 mM MgCl_2_ × 6H_2_O, 4.7 mM KCl, and 132 mM NaCl_2_ (pH 7.4)] to destabilize the alginate crosslinks. Samples were then rinsed twice in the mannitol buffer for 2 min each. The samples were then incubated in the PBAA coating solution for 15 to 20 min with mild agitation. The process was monitored by phase contrast microscopy (Nikon), through which the formation of a thin coating could be observed. Coated samples were then rinsed three times with normal saline before culturing in media or storage in saline supplemented with 2 mM CaCl_2_, in the case of acellular samples.

### Cryogel porosity measurements

Two-dimensional porosity measurements resulted from comparing the average wall thickness and average pore size measured by image analysis of SEM images of cryogel skeletons.

Three-dimensional porosity measurements involved imbibing cryogels with methanol, which minimally swells silicone, following the pre-filling protocol described in the fabrication of cryogel composites. The methanol-filled weight of the cryogel was then measured using a microbalance (Mettler Toledo). The porosity *V_p_* was then calculated using the following equation

$$V_{p}=\frac{m_{\text{fill}}-m_{\text{dry}}}{V\;*\;d_{\text{MeOH}}}$$

where *m*_fill_ (in milligrams) is the mass of the sample filled with methanol, *m*_dry_ (in milligrams ) is the dry weight of the sample, *V* (in cubic millimeters) is the volume of the sample, and *d*_MeOH_ is the density of methanol (0.792 mg/μl).

### Contact angle measurements

Static contact angle images were captured and analyzed using a Krüss drop shape analyzer. All measurements used a 5-μl drop of deionized water.

### 3D cell density quantification

Cell-laden samples were cut into fine pieces using sterile stainless-steel scissors immediately before cell population quantification using a luminescent cell viability assay designed for 3D microtissue cultures (Promega, catalog no. G9681). Standards with known cell populations were used to convert luminescence values to cell counts. The cell counts were then normalized by the measured dimensions of samples to calculate the effective cell density of samples.

### Analysis of cellular spatial distribution

Fluorescently stained cell-laden composites were first sectioned with a razor blade and then placed cut side down in a glass-bottom well plate before confocal microscopy. The resulting images were then manually cropped to the size of the sample before processing by a Python (version 3.11.1) image analysis program based on the OpenCV computer vision library. The algorithm used a quadrat-based method for quantifying the spatial distribution of particles (*65*). Briefly, the algorithm applies a binary threshold, divides the image into a grid of equally sized squares, and then counts the number of cells in each square using contour detection. The resulting data are then used to calculate the average number of cells per square as a metric for cell density, as well as the index of skewness as a metric for distribution uniformity. The index of skewness *S* was calculated using the following formula

$$S=\frac{q}{\left(q-1\right)\left(q-2\right)}\sum_{i\;=\;1}^{q}{\left(\frac{x_{i}-\bar{x}}{\sigma }\right)}^{3}$$ (1)

where *q* is the total number of squares, *x_i_* is the number of cells in square *i*, $\bar{x}$ is the average cell count, and σ is the SD of counts.

### Mechanical testing

Mechanical characterization used an Instron Universal Testing Machine. Compression testing was performed at a rate of 0.015 mm/s, while tensile elongation occurred at a rate of 0.1 mm/s. Compression testing used cylindrical metal platens. For tensile testing, cyanoacrylate glue (Krazy Glue) facilitated the bonding of gelled samples to custom-made acrylic fixtures which fit snugly into clamping tension grippers.

### Diffusion measurements

A side-by-side diffusion cell with a 10-ml volume acceptor and donor chambers and a 1-cm orifice (PermeGear Inc) was used to measure the transport properties of various materials. A magnetic stirring plate underneath the diffusion cell coupled with magnetic stir bars in each chamber ensured homogeneous analyte concentrations. All measurements occurred at RT. Calculation of Fickian diffusivity *D* used the following equation

$$D=\frac{m*V*t}{A*∆c}$$ (2)

Here, *m* (in moles per cubic centimeter per second) is the linear slope of the concentration-versus-time plot for a given analyte, *V* (in cubic centimeters) is the volume of the acceptor chamber, *t* (cm) is the thickness of the sample, *A* (in square centimeters) is the cross-sectional area of the orifice, and ∆*c* (in moles per cubic centimeter) is the difference in analyte concentration between the two sides of the diffusion cell.

For oxygen diffusivity measurements, samples were deoxygenated immediately before measurement. The deoxygenation process involved sealing samples immersed in deionized water inside a glass vial sealed with a rubber septum and electrical tape before bubbling with argon gas for 45 min. The same process was used to prepare deoxygenated water. The donor chamber of the cell was filled with deionized water equilibrated with the atmosphere. The acceptor chamber was sealed with a rubber septum and before receiving an injection of deoxygenated water through a 16G needle. A second 16G needle served as an outlet for gas displaced by the deoxygenated water and was immediately removed once the chamber was filled. A coaxial fiberoptic oxygen probe (PreSens Precision Sensing GmbH) injected into the deoxygenated water through a syringe needle monitored the dynamic oxygen concentration.

Measurement of macromolecular diffusivity began by preparing a solution (10 mg/ml) of FITC-dextran (average MW: 4000 Da) in water. After placing samples in the orifice, the donor chamber received the FITC-dextran solution and the acceptor chamber received deionized water. At regular time intervals, 200-μl aliquots were taken from the acceptor chamber and replaced with an equivalent volume of water. The absorbance at 495 nm was measured for each aliquot and converted to a concentration using a freshly prepared standard curve.

### Transformation and maintenance of cell lines

HEK-292T cells were cultured in Dulbecco’s modified Eagle’s medium (high glucose) supplemented with 10% fetal bovine serum and 1% penicillin-streptomycin at 37°C with 5% CO_2_. Transgenic HEK cell lines secreting either G-Luc or mouse EPO were transformed using an engineered lentivirus following a previously published protocol (*29*).

### In vitro protein secretion rate measurements

Samples containing encapsulated HEK-GLuc cells were rinsed twice with DPBS before incubation in 2 ml of media. After 1 hour, an aliquot of the supernatant was collected and analyzed using a G-Luc assay kit. Mouse EPO secretion rate measurements followed a similar protocol except samples were incubated overnight before quantification by ELISA.

### DNA extraction and quantification

Cell-laden samples were flash-frozen by plunging them into liquid nitrogen. Frozen samples were then wrapped with weighing paper and dipped into liquid nitrogen to maintain their brittleness. Samples were then carefully fractured and transferred back into cooled microtubes. DNA was then extracted from samples using a commercial kit (Qiagen). Samples were eluted twice from the purification column to ensure maximum yield. The isolated DNA was then quantified using a spectrophotometer calibrated for nucleic acid detection (DeNovix).

### Implantation and retrieval in mice

All animal procedures were approved by the Massachusetts Institute of Technology (MIT) Committee on Animal Care and supervised by the MIT Division of Comparative Medicine veterinary staff. BALB/c and C57BL/6 mice of 6 to 8 weeks of age were obtained from the Jackson Laboratory. Animals were anesthetized with 3% isoflurane in oxygen. To prepare for surgery, the surgical site was shaved and sterilized using betadine and isopropanol. Buprenorphine SR-LAB (0.03 mg; ZooPharm) and 1 ml of 0.9% saline were administered subcutaneously for analgesia and hydration.

For subcutaneous surgeries, a small incision was made along the back of mice. A pocket under the skin large enough to comfortably accommodate 7-mm-diameter hydrogel disks was created by blunt dissection with a hemostat. One implant was then inserted on each side of the spine before closing the incision using wound clips.

For intraperitoneal surgeries, a small incision was made along the midline of the abdomen, and a blunt dissection exposed the peritoneal lining. Another incision along the linea alba exposed the peritoneal cavity where implants were carefully inserted away from the fat pad. The peritoneum incision was closed using absorbable Vicryl sutures (Ethicon Inc.). The skin incision was closed using wound clips.

For retrieval, mice were euthanized by CO_2_ inhalation and cervical dislocation. Opening the peritoneal cavity allowed implants to be located and transferred to sterile saline containing calcium chloride. Retrieved implants were then cultured overnight before ex vivo analyses.

### Monitoring of serum protein dynamics in mice

A total of 100 to 200 μl of blood was collected from either the saphenous or femoral vein of mice in a blood collection tube containing a clot activator and serum gel separator (BD

Biosciences, catalog no. 365967). The tubes were inverted several times to help facilitate clotting before placing them on ice. Tubes were then centrifuged at 10,000 RCF for 10 min in a cold room to isolate the blood serum. The collected serum was transferred to clean microtubes before proceeding to protein quantification by either G-Luc assay or mouse EPO ELISA.

### Measurement of mouse hematocrit levels

Blood samples collected from mice after 8 weeks of treatment were fractionated by centrifugation at 10,000 RCF for 10 min in clear blood collection microtubes. The volume percentage of the red blood cell fraction in each sample was then determined by digital image analysis using ImageJ.

### Bioluminescence characterization of implants containing firefly luciferase–expressing HEK-EPO cells

After measuring protein secretion rates, implants were rinsed with DPBS before incubating in a luciferin solution (0.5 mg/ml) prepared in Kreb’s buffer containing calcium chloride for 25 min. The implants were then imaged and analyzed using an IVIS Spectrum imaging system (PerkinElmer).

### Fibrosis evaluation on explanted implants

Following all other ex vivo characterizations, explanted samples were fixed in 10% formalin for 45 min and embedded in low–melting point agarose to reinforce any fibrotic tissue on the outer surface before sectioning in two with a sharp razor blade. One section was used for collagen content determination, and the other section was used for histological analysis.

Collagen content determination used a hydroxyproline assay. Bright-field stereoscopic imaging enabled precise surface area measurement of each section. Sections were then boiled in 6 M HCl at 120°C for at least 3 hours to hydrolyze the collagen. The resulting solution was used for the assay, and the surface area, obtained from image analysis of bright-field micrographs, was used to calculate the areal density of surface collagen of each section.

Histology was performed at the Hope Babette Tang Histology Facility at the Koch Institute at MIT. After embedding in agarose and sectioning, samples were dehydrated in ethanol, and then processed for paraffin embedding where care was taken to orient the samples such that histological sections of the implant cross sections could be obtained. To evaluate connective tissue surrounding implants, samples were sectioned using a microtome, stained using Mason’s trichrome stain, and scanned with a digital pathology slide scanner (Leica). Subsequently, trichrome-stained sections were further analyzed using ImageJ software for quantification of average fibrotic tissue thickness. For each sample, six to seven measurements were made at different locations along the thickness of the dark blue band of tissue and then averaged.

Additional histological sections for IHC were treated with citrate buffer pH 6 at 97°C for 20 min for antigen retrieval before automated IHC staining (Thermo Fisher Scientific). Immunostaining involved endogenous peroxidase and protein blocking followed by 60 min of incubation with either anti-mouse α–smooth muscle actin (ABT1492, Sigma-Aldrich) 1:800 dilution or F4/80 (70076, Cell Signaling Technologies) 1:2000 dilution. Slides were then labeled with peroxidase-conjugated secondary antibodies and developed for 5 min with diaminobenzidine to generate a dark brown chromogen at target cells. Slides were then counterstained with hematoxylin before digital scanning.

### Statistics

Details regarding sample size and appropriate statistical tests are described in the figure captions or main text. The data are expressed as means ± SE. Statistical conclusions resulted from either unpaired, two-tailed *t* tests or one-way analysis of variance (ANOVA) using the Holm-Bonferroni method for multiple comparison correction calculated using OriginPro 2023. Statistical significance followed the following convention: *P < 0.05, **P < 0.01, and ***P < 0.001.
