# Supplementary material for: Silicone cryogel skeletons enhance the survival and mechanical integrity of hydrogel-encapsulated cell therapies
Source: Sci Adv. 2024 Apr 5;10(14):eadk5949. doi: 10.1126/sciadv.adk5949 (PMC10997197; doi:10.1126/sciadv.adk5949)
Supplement: Supplementary file 1 — Figs. S1 to S22 Table S1 Legends for movie S1 [file sciadv.adk5949_sm.pdf]

Science Advances
AAAS

Supplementary Materials for

Silicone cryogel skeletons enhance the survival and mechanical integrity of hydrogel-encapsulated cell therapies

William J. Jeang *et al.*

Corresponding author: Daniel G. Anderson, dgander@mit.edu

Sci. Adv. **10**, eadk5949 (2024)
DOI: 10.1126/sciadv.adk5949

The PDF file includes:

Figs. S1 to S22
Table S1
Legends for movie S1

Other Supplementary Material for this manuscript includes the following:

Movie S1

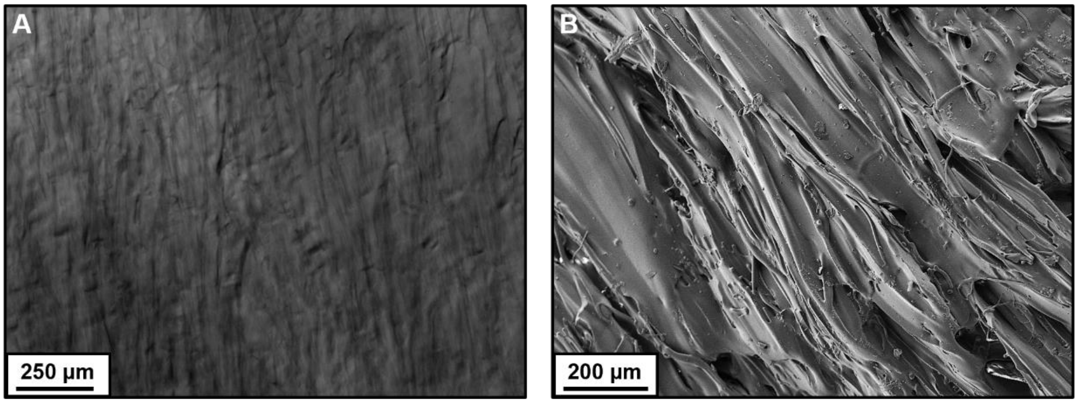

Fig. S1. Microstructure of tert-butanol silicone cryogels. (A) Brightfield micrograph of cryogels synthesized using tert-butanol as a solvent, soaked with ethanol. **(B)** Scanning electron micrograph of dried cryogels highlighting the fibrous microstructure.

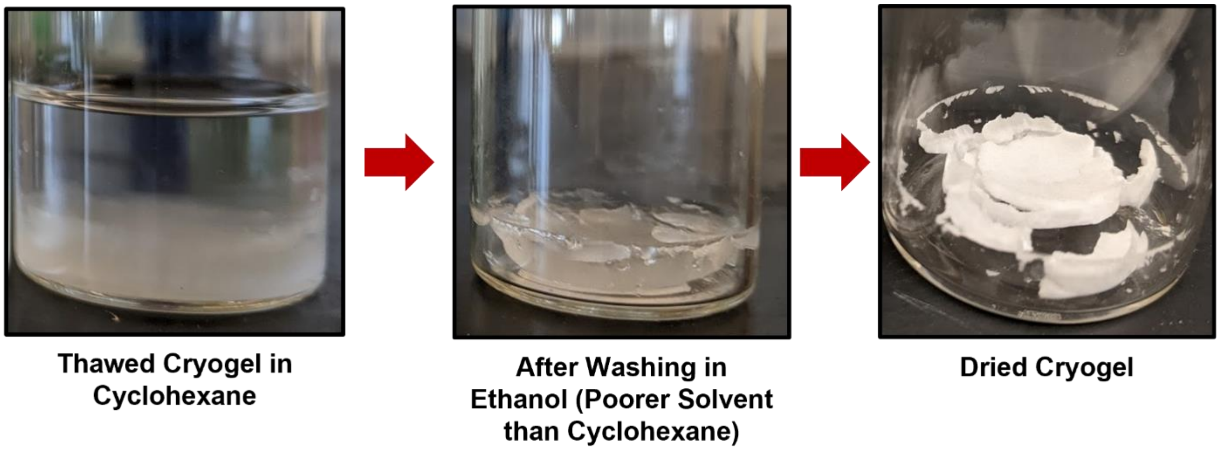

Fig. S2. Brittleness of unoptimized cryogel formulation. Photographs of the deswelling and drying process showing spontaneous fracturing.

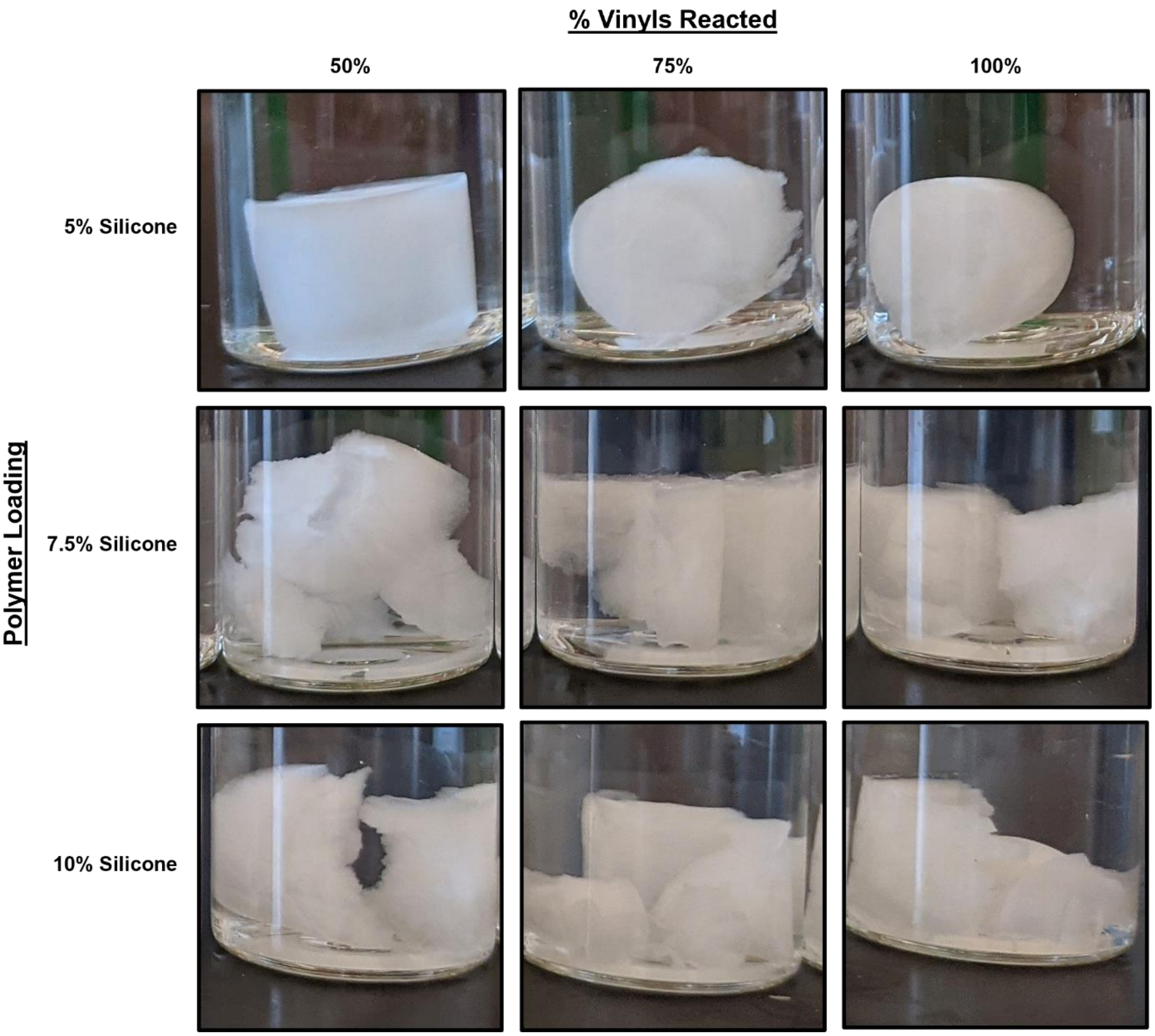

Fig. S3. Composition optimization to achieve resilient silicone cryogel skeletons. Photographs of de-swollen cryogels formulated using different concentrations of silicone and dithiol crosslinker. Samples with 5% silicone were able to withstand deswelling and removal from the reaction vessel.

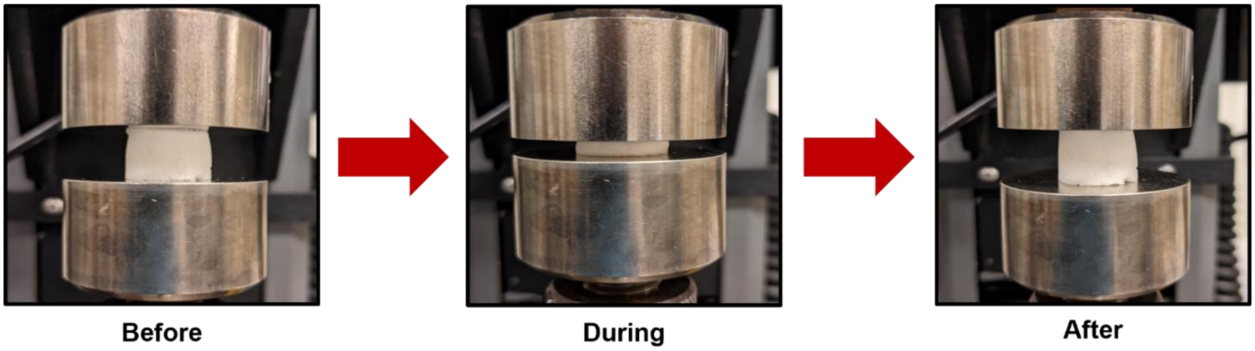

Fig. S4. Resiliency of optimized cryogel skeleton. Photographs of a dry cryogel skeleton exhibiting elastic recovery after high-strain compression.

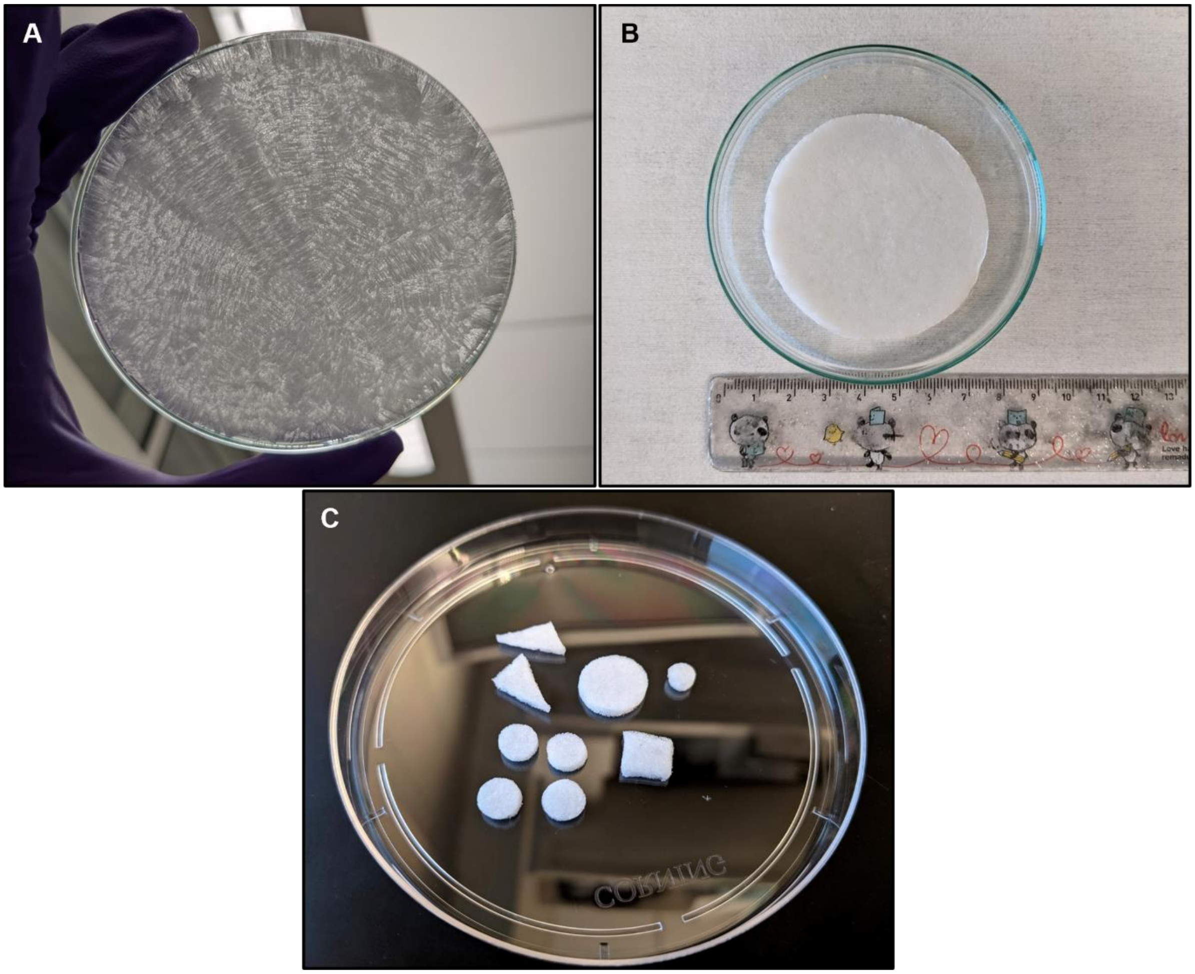

Fig. S5. Facile fabrication of varying device geometries. Photographs of cryogel skeletons cast to a desired thickness after **(A)** thawing, **(B)** deswelling and drying, and **(C)** cutting into various shapes.

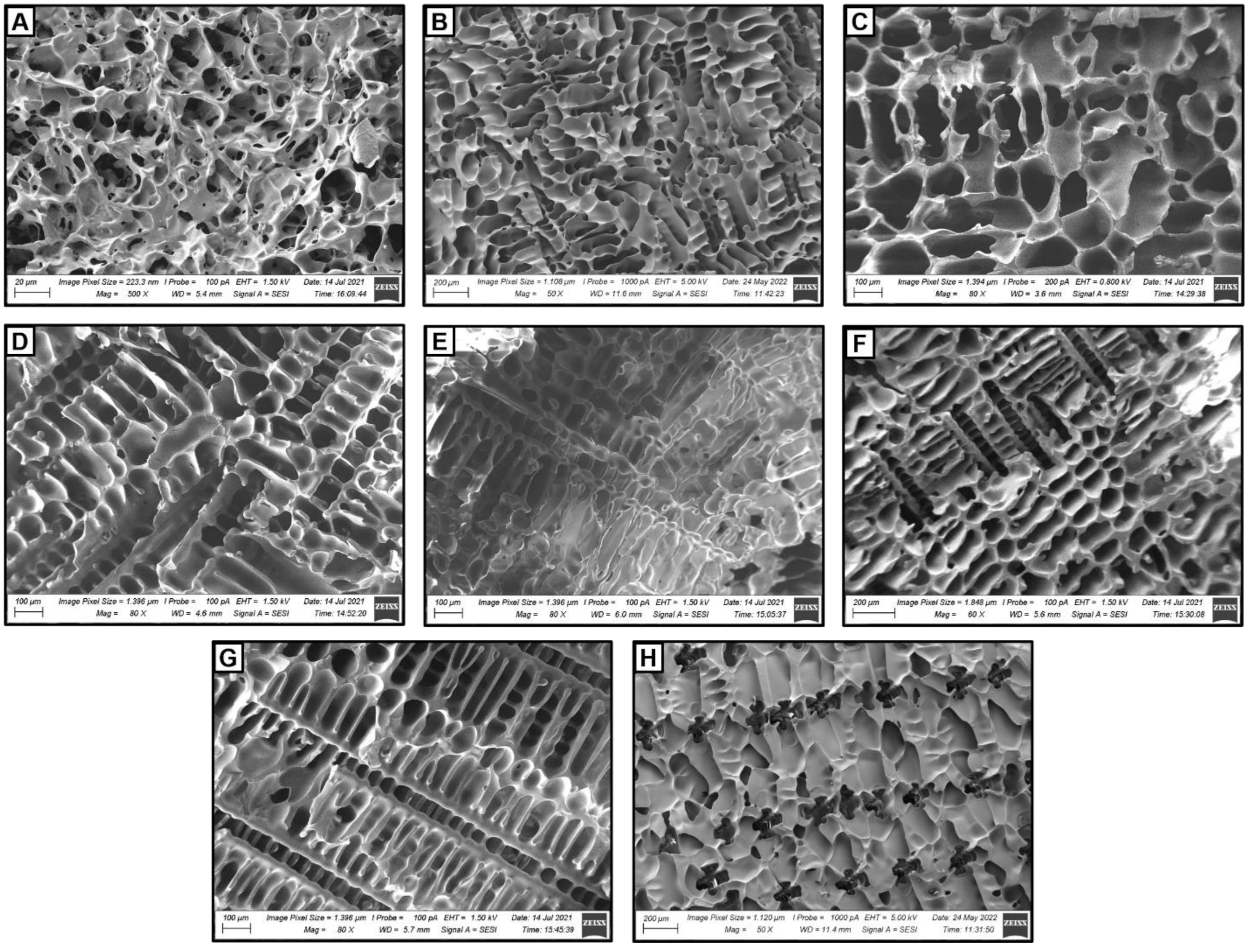

Fig. S6. Diverse skeleton microstructures achieved using different compositions and freezing conditions. SEM micrographs of the microstructures corresponding to synthetic conditions detailed in Table S1.

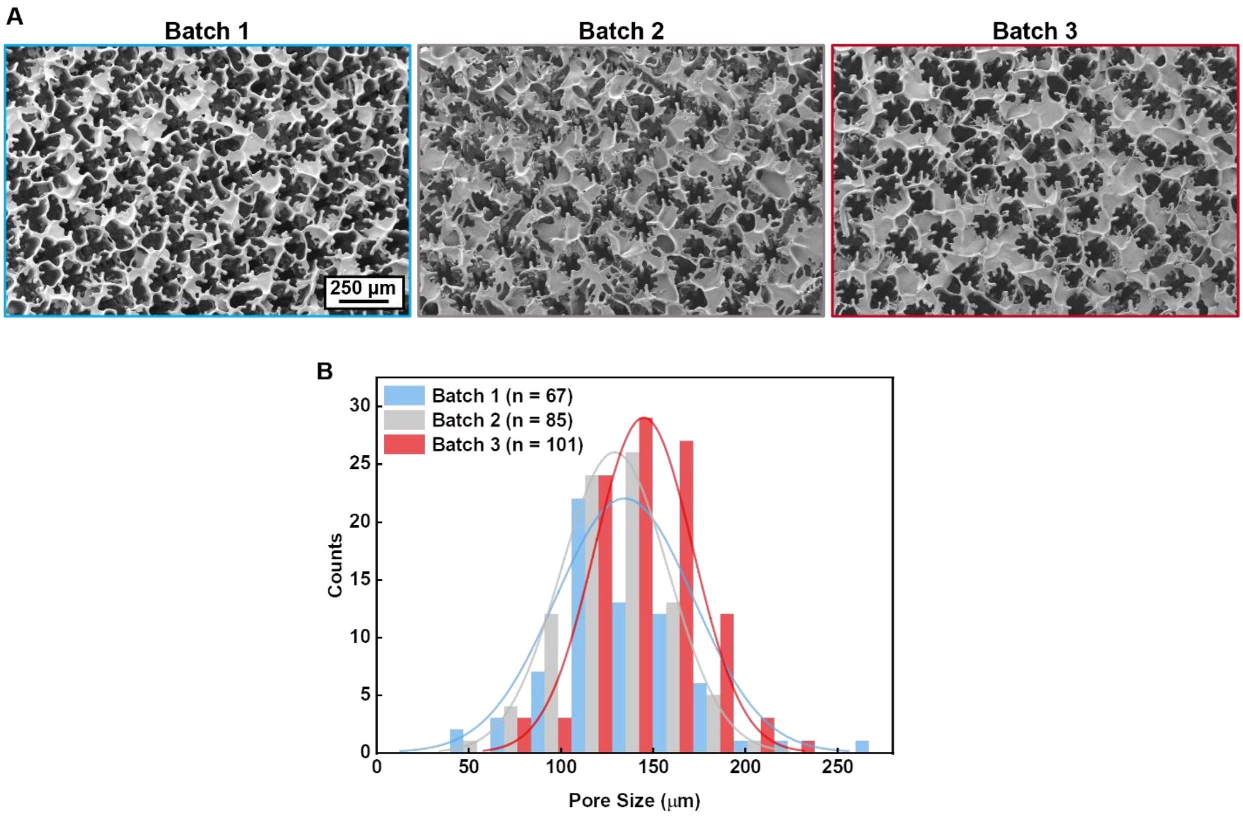

Fig. S7. Batch-to-batch variation in the microstructure of optimized skeletons. (A) SEM images of 3 separately synthesized batches of cryogel skeletons. Batch 1 is the same batch as depicted in Fig. 2B. **(B)** Histograms and overlayed normal fitted curves of pore size measurements taken from each batch. Average pore size +/- SD: Batch 1 = 135 +/- 37 µm, Batch 2 = 129 +/- 29 µm, Batch 3 = 145 +/- 27 µm.

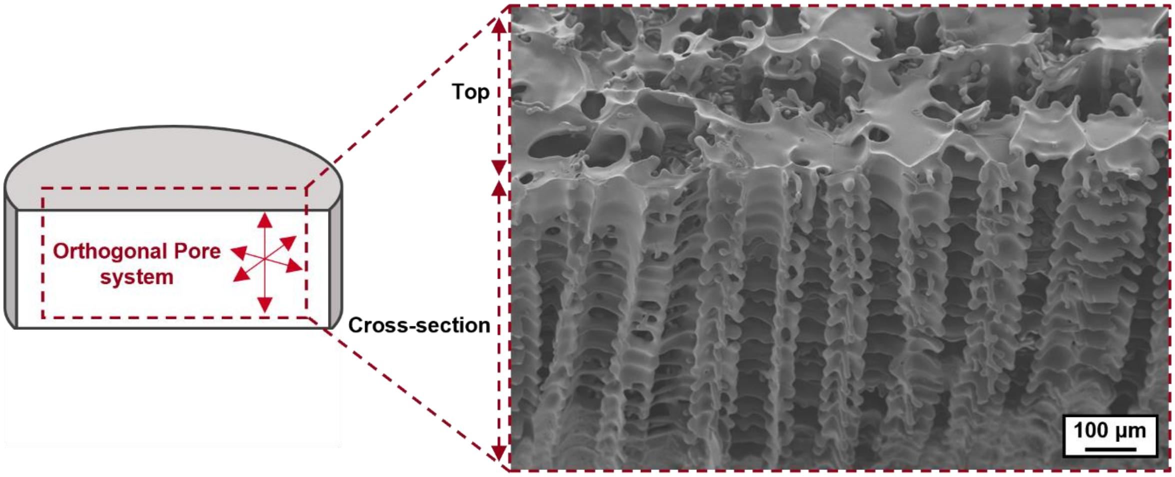

Fig. S8. Cross section of orthogonal pore system. SEM image of sectioned skeleton showing straight channels extending down from the top surface with regularly spaced lateral interconnects.

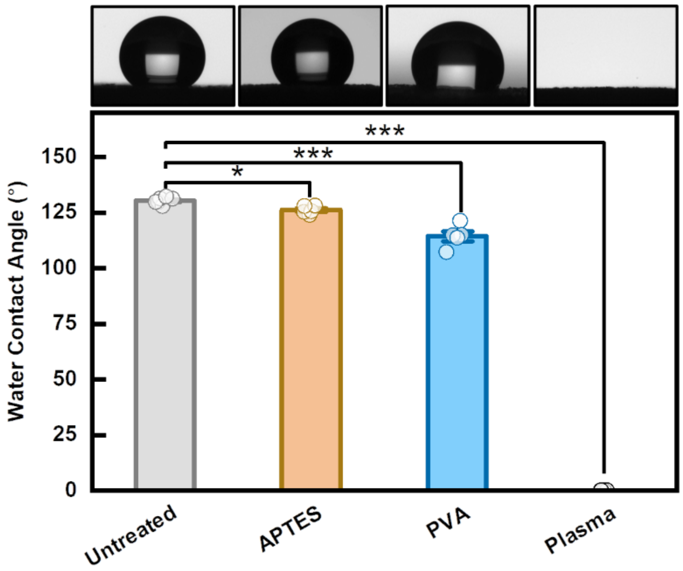

Fig. S9. Hydrophilization of silicone cryogel skeletons. Water contact angle data of cryogels treated with different hydrophilic materials or after air plasma treatment. Photographs of sessile water droplets for each condition displayed above.

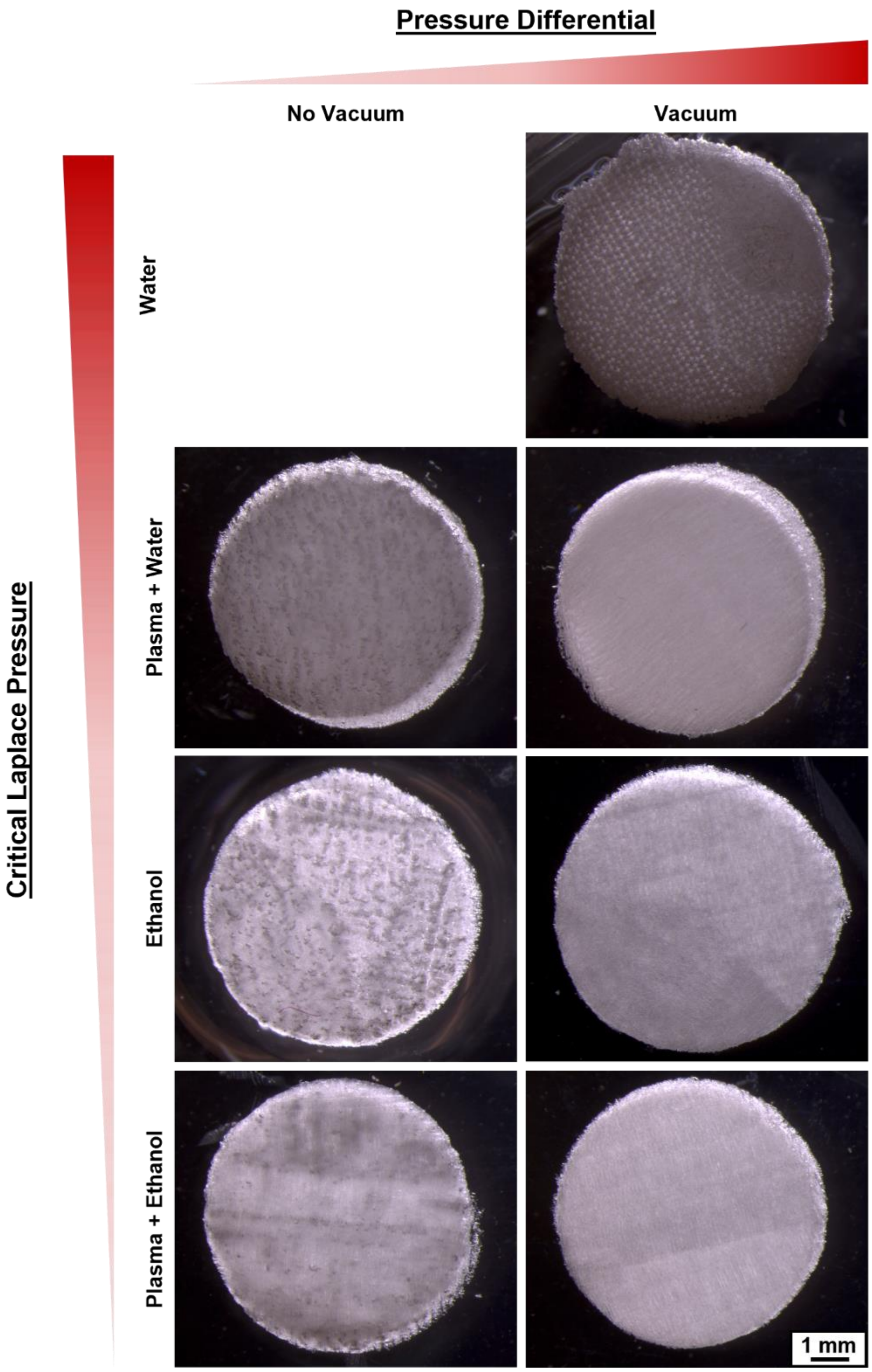

Fig. S10. Fluid infiltration of superhydrophobic silicone cryogel skeletons. Brightfield microscope images of cryogel skeletons infiltrated to varying degrees using infiltration methods based on modulating the critical Laplace pressure or applying an external pressure differential. Samples treated with only water completely rejected infiltration and remained floating atop the water surface, even when vacuum was applied. Plasma treatment and ethanol pre-wetting led to partially filled samples with numerous air bubbles. Combination approaches left no visible bubbles.

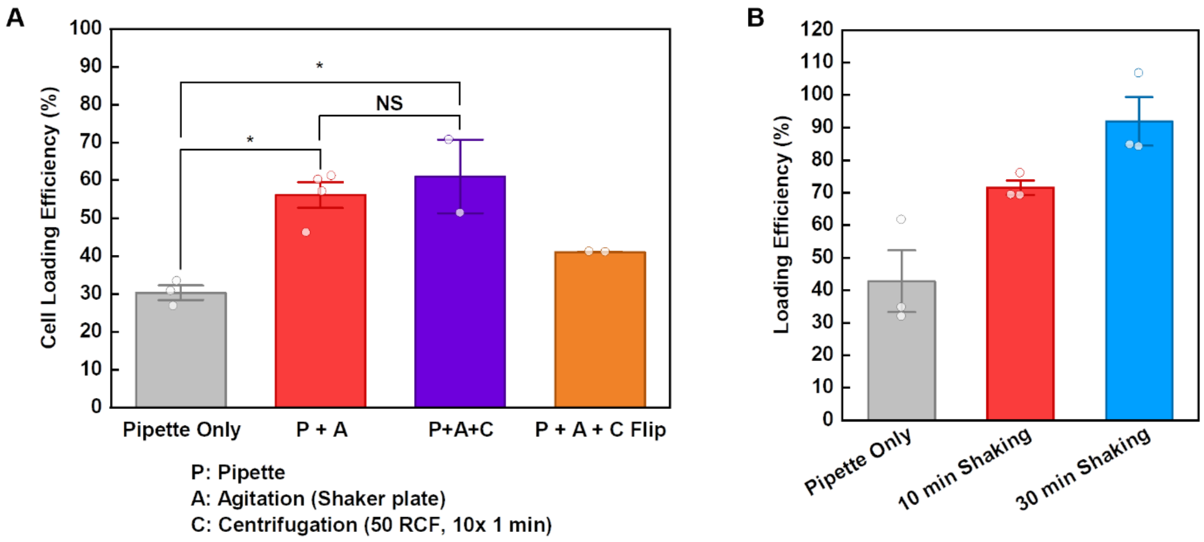

Fig. S11. Cell loading optimization. (A) Plot comparing the efficiencies of different active cell loading methods. Samples with agitation were placed on an orbital shaker plate for 10 minutes at 500 RPM. Centrifuged samples underwent 10 1-minute cycles at 50 RCF. Flip samples were flipped over after each cycle of centrifugation. Significance was determined by one-way ANOVA using the Holm-Bonferroni method for multiple comparison correction (*$P < 0.05$). **(B)** Plot showing improved loading efficiency with longer agitation time. Error bars for both plots indicate SE.

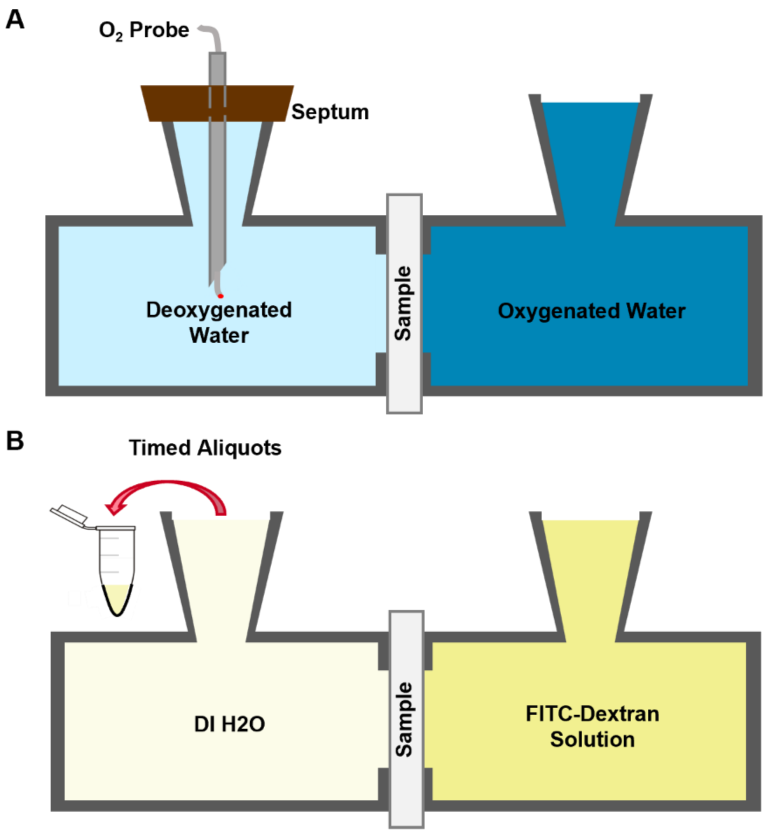

Fig. S12. Diffusivity measurement setup. Schematics illustrating the setups used for measuring the diffusivity of **(A)** oxygen and **(B)** fluorescently labeled dextran polymer (FITC-Dextran). A fiberoptic oxygen probe provided real-time oxygen readings. Changes in FITC-Dextran concentration in the DI water chamber were obtained by comparing the fluorescence readings of aliquots taken at timed intervals against a standard curve.

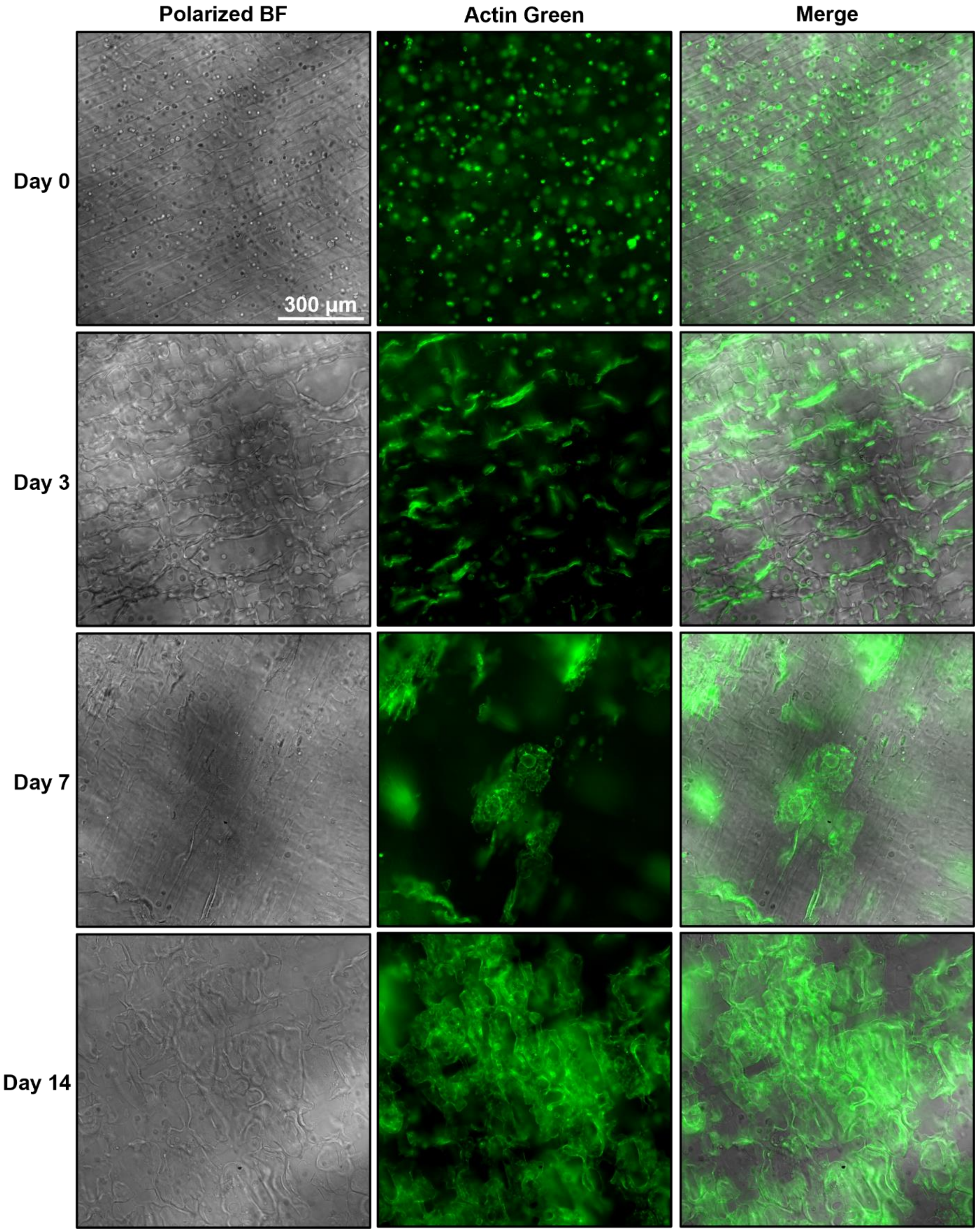

Fig. S13. Cell scaffolding in skeletal composites. Compilation of polarized brightfield and epifluorescence images of fluorescent HEK cells cultured for varying amounts of time at 5% oxygen showing a transition from dispersed round cells to asymmetric clusters along the skeleton.

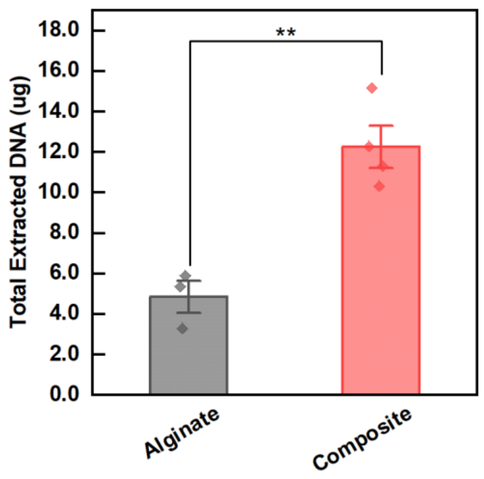

Fig. S14. Quantification of DNA extracted from samples after 2 weeks of low oxygen culture. Statistical significance was calculated by an unpaired, two-tailed t-test *(**P* < 0.01).

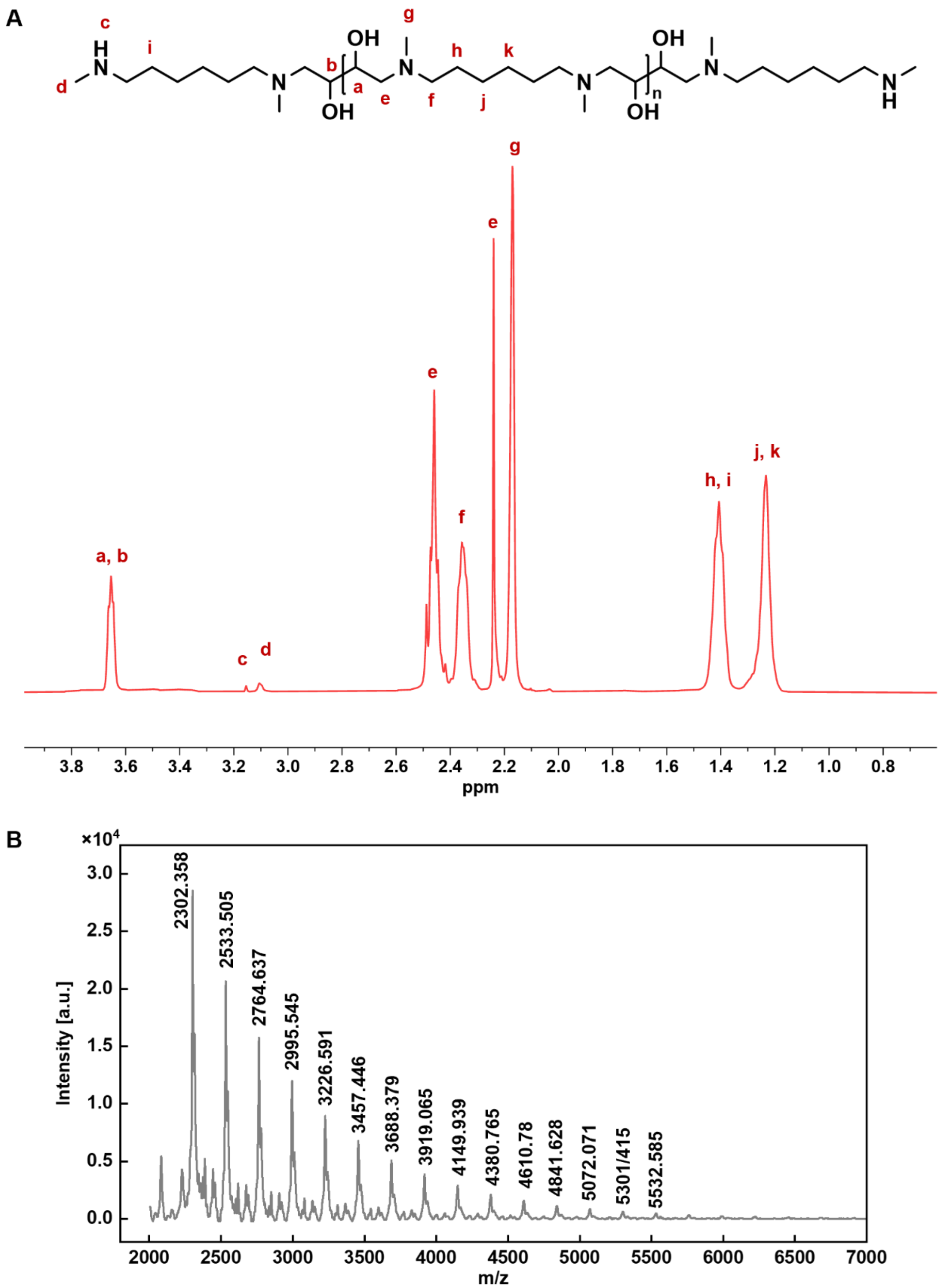

Fig. S15. Synthesis confirmation and molecular weight determination of the PBAA. (A) NMR spectrum and **(B)** MALDI-TOF mass spectrometry data of the PBAA.

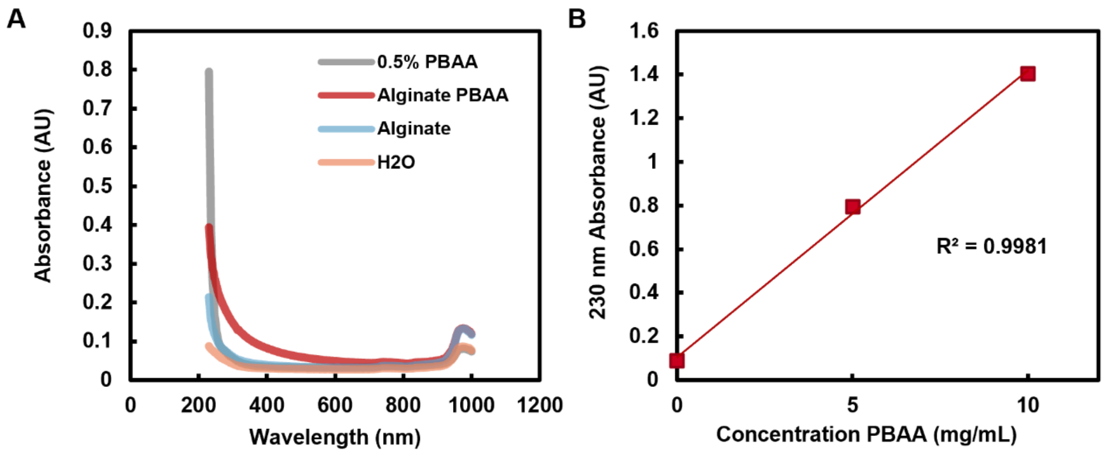

Fig. S16. PBAA Coating validation by UV-VIS spectroscopy. (A) UV-VIS spectra of PBAA, alginate with and without PBAA, and water. **(B)** 230 nm light absorbance of solutions with increasing concentration of PBAA.

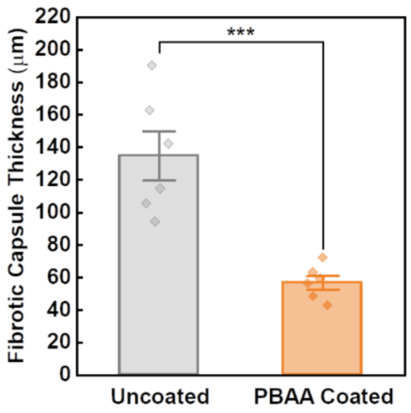

Fig. S17. Quantification of fibrotic capsule thickness. Measurements taken by image analysis of histological samples following Masson's Trichrome staining. Statistical significance was calculated by an unpaired, two-tailed t-test *(***P* < 0.001).

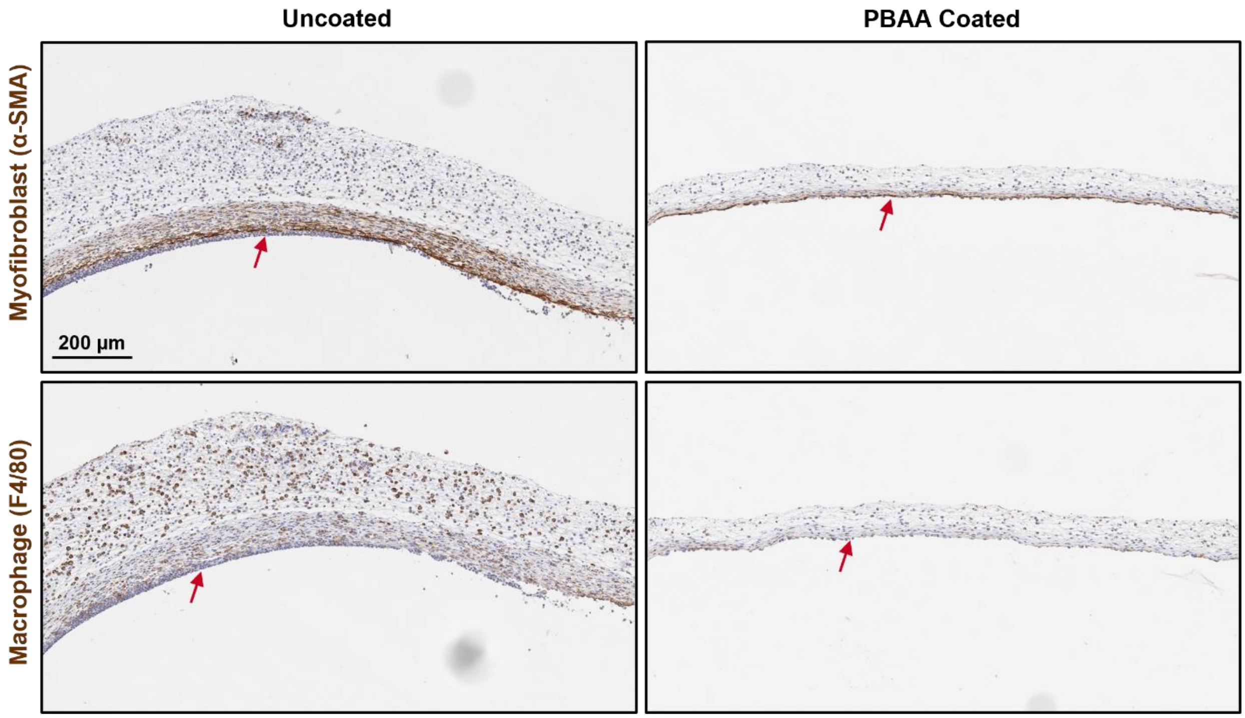

Fig. S18. Immunohistochemistry of fibrosis on subcutaneous alginate implants. Histological sections of retrieved implants with (right) and without (left) a PBAA coating. Sections were stained by DAB IHC (Brown) using antibody targets for α-smooth muscle actin positive myofibroblasts (top) and F4/80 positive macrophages (bottom). All sections were counterstained with a hematoxylin nuclear stain (blue). Red arrows indicate implant interface on the inferior side of the tissue section.

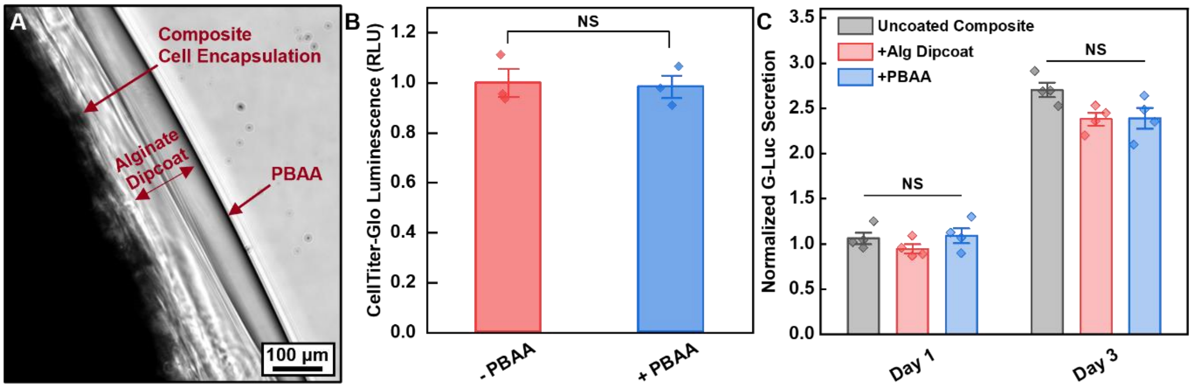

Fig. S19. Effect of alginate and PBAA coatings on viability and secretion. (A) Optical micrograph of composite-encapsulated HEK cells dip coated with acellular alginate (1.4 wt%) and further coated with PBAA. **(B)** Plot showing minimal loss of viability in samples immediately following the PBAA coating process, as measured by CellTiter-Glo 3D assay. Statistical significance was determined by an unpaired, two-tailed t-test. **(C)** Measurements of Gaussia luciferase (G-Luc) secretion from samples with either no coating, an acellular alginate dipcoat, or an alginate coat with the PBAA. Samples were incubated at 5% oxygen. Secreted G-Luc luminescence was normalized by measurements from uncoated samples on Day 1. Significance was determined one-way ANOVA using the Holm-Bonferroni method for multiple comparison correction.

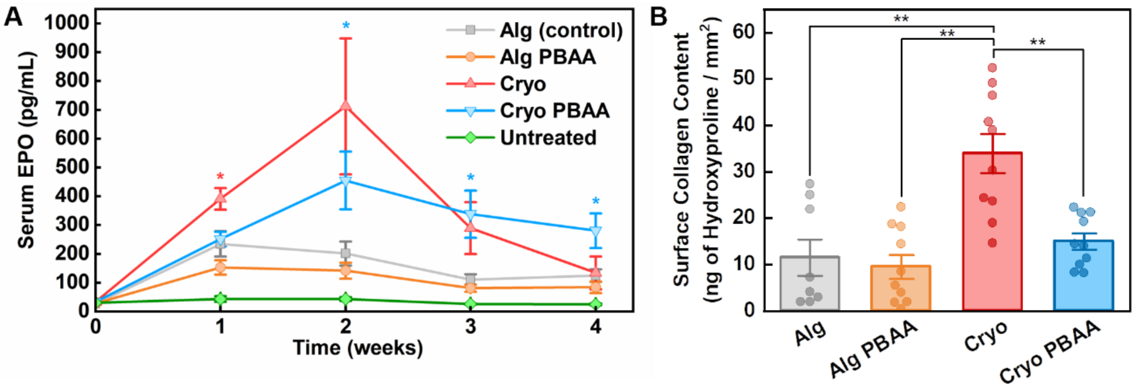

Fig. S20. Initial in vivo study with HEK-EPO-containing implants coated with 1% alginate. **(A)** Plot of EPO levels in mouse serum over 4 weeks showing sustained elevated protein levels with PBAA coated cryogel composites. Week 0 time points represent basal serum EPO levels in mice with no implants. Alg: alginate (n = 9), Alg PBAA: PBAA-coated alginate (n = 10), Cryo: Silicone cryogel composite (n = 10), Cryo PBAA: PBAA-coated composite (n = 10), Untreated (n = 5). Statistical significance was determined for the comparison of each test group against alginate at each time point from unpaired, two-tailed t-tests. Asterisk colors correspond with the colors of the significant test groups. **(B)** Surface collagen concentrations quantified by hydroxyproline assay of proteins extracted from samples. Significance was determined by one-way ANOVA using the Holm-Bonferroni method for multiple comparison correction *(**P* < 0.01).

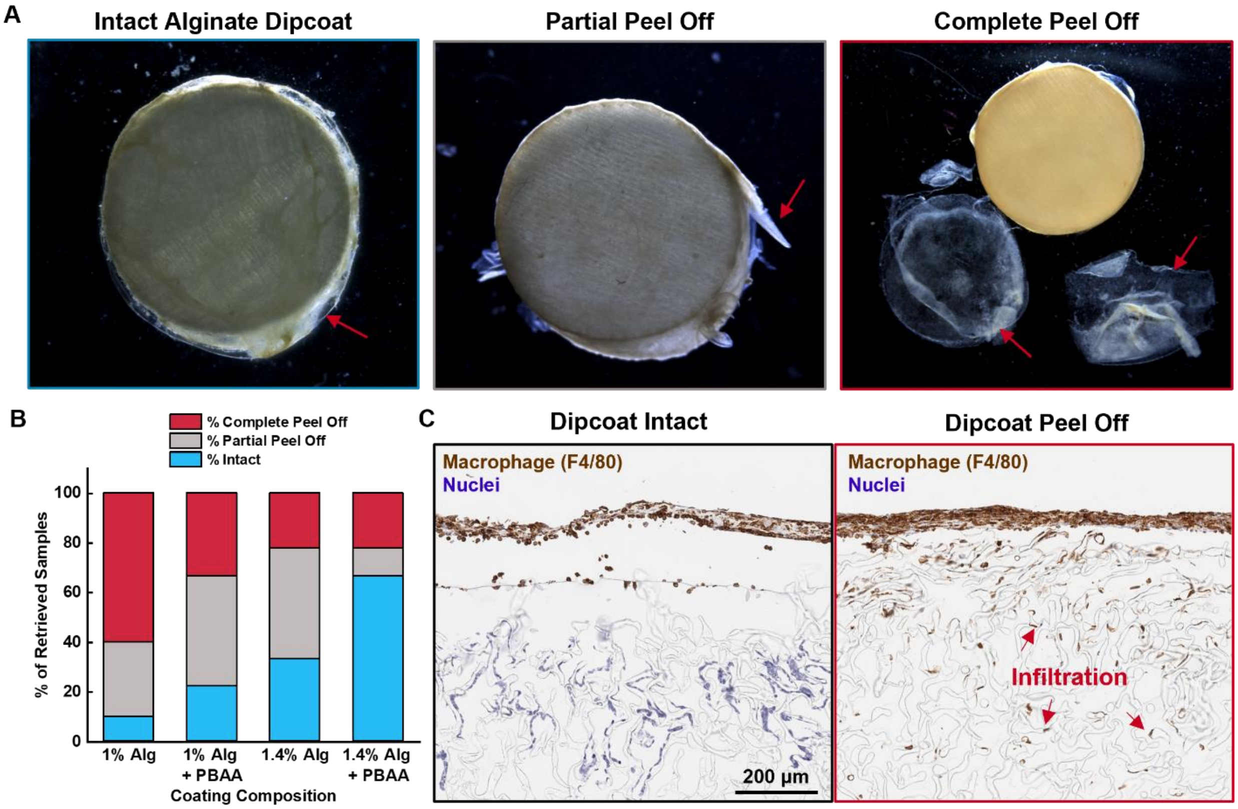

Fig. S21. Effect of coating composition on mechanical failure and immune cell infiltration in vivo. (A) Micrographs of the varying states of the acellular alginate dipcoat observed on retrieved composite encapsulations. Red arrows indicate the alginate hydrogel coating. **(B)** Plot summarizing the effect of coating composition on the integrity of coatings following implantation. Results represent data collected from several different animal experiments with implantation durations ranging from 4 to 8 weeks. Sample sizes: 1% Alg (n = 10), 1% Alg + PBAA (n = 9), 1.4% (n = 9), 1.4% + PBAA (n = 9). **(C)** Histological sections stained by IHC showing confinement of macrophages to the surface of composite implants with intact alginate dipcoats (left), and macrophage infiltration into the cryogel skeleton (transparent network with grey outline) in the samples with ablated dipcoats (right). All sections were counterstained with a blue hematoxylin nuclear stain, revealing encapsulated cells inside skeletons with intact dipcoats.

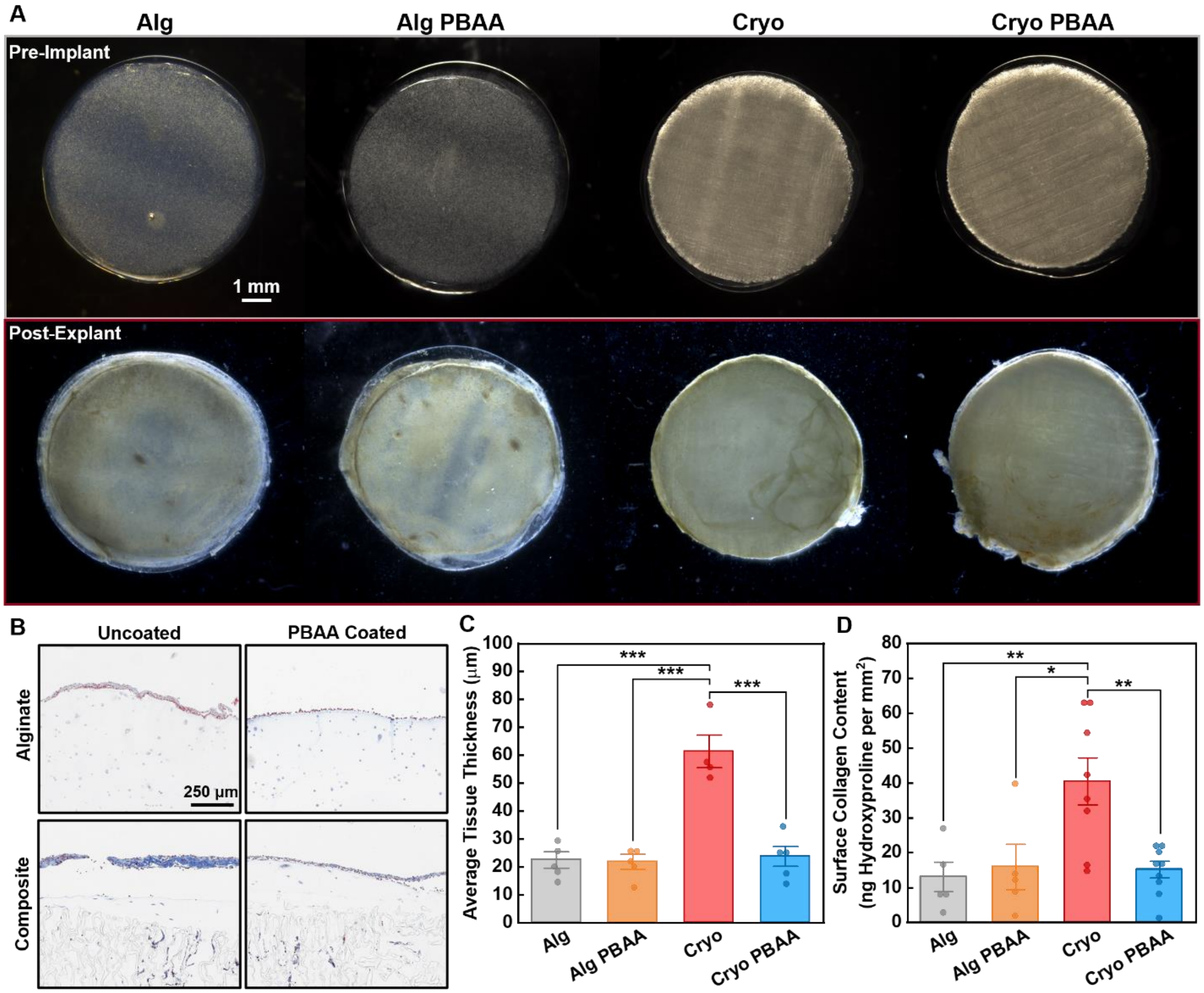

Fig. S22. Fibrosis of retrieved implants. (A) Micrographs of samples before (top row) and after (bottom row) intraperitoneal implantation in mice for 8 weeks. **(B)** Histological cross sections stained with Masson's trichrome stain. The alginate phase in both alginate and composite samples are lightly stained pale blue, while cell nuclei and fibrotic tissue appear black and dark blue, respectively. **(C)** Quantification of the average tissue thickness from trichrome-stained sections. **(D)** Comparison of surface collagen content, measured by hydroxyproline assay of proteins extracted from samples, showing elevated fibrosis on cryogel composite samples but not on PBAA-coated composites. For all plots, error bars indicate SE and significance was computed by one-way ANOVA using the Holm-Bonferroni method for multiple comparison correction (*$P < 0.05$, **$P < 0.01$, ***P, < 0.001).

SEM Image Panel	Silicone Content (%)	Vinyl Group Crosslinking (%)	Freezing Temperature (°C)	Insulation	Major Axis Pore Size (µm ± SD)	Minor Axis Pore Size (µm ± SD)	Average Pore Size (µm ± SD)	Compressive Modulus (kPa)
**A**	5	50	-196	No	10.5 ± 4.3	5.4 ± 2.6	8.0 ± 4.4	-
**B**	5	75	-18	No	153.2 ± 50.5	68.9 ± 20.4	111.1 ± 57.1	-
**C**	5	50	-18	Yes	194.2 ±82.6	92.0 ± 26.5	143.1 ± 79.8	7.93
**D**	5	75	-18	Yes	140.7 ± 54.5	61.3 ± 16.3	101.0 ± 56.5	12.2
**E**	5	100	-18	Yes	155.4 ± 50.3	51.7 ± 9.9	103.5 ± 63.3	18.8
**F**	7.5	75	-18	Yes	188.4 ± 66.1	80.6 ± 16.9	134.5 ± 72.3	-
**G**	10	75	-18	Yes	117.1 ± 55.4	51.3 ± 13.9	84.2 ± 52.1	-
**H**	5	75	-12	Yes	-	-	159.6 ± 30.2	16.1

Table S1. Synthesis conditions, mechanics, and quantitative pore size analysis for skeletons shown in Fig. S6. Samples frozen at -196 ℃ utilized liquid nitrogen. All other samples were frozen in a controlled freezer. Samples with insulation were insulated by surrounding the glass reaction vessel with Styrofoam. Pore size measurements were performed using ImageJ.

Movie S1. 3D-reconstruction from confocal microscopy of quantum dot-laden cryogel (magenta) encapsulating cells treated with Calcein AM live stain (green) after 2 weeks in culture at 5% O2. The dimensions of the reconstructed volume are 1440 µm x 1080 µm x 80 µm.
